# Azobenzene-based sinusoidal surface topography drives focal adhesion confinement and guides collective migration of epithelial cells

**DOI:** 10.1038/s41598-020-71567-w

**Published:** 2020-09-18

**Authors:** Chiara Fedele, Elina Mäntylä, Brian Belardi, Tiama Hamkins-Indik, Silvia Cavalli, Paolo A. Netti, Daniel A. Fletcher, Soile Nymark, Arri Priimagi, Teemu O. Ihalainen

**Affiliations:** 1 Faculty of Engineering and Natural Sciences, Tampere University, Tampere, Finland; 2 BioMediTech and Faculty of Medicine and Health Technology, Tampere University, Tampere, Finland; 3 Department of Bioengineering and Biophysics Program, University of California, Berkeley, CA 94720 USA; 4 Istituto Italiano Di Tecnologia, Center for Advanced Biomaterials for Healthcare @CRIB, Naples, Italy; 5 Chan Zuckerberg Biohub, San Francisco, CA 94158 USA; 6 Division of Biological Systems and Engineering, Lawrence Berkeley National Laboratory, Berkeley, CA 94720 USA

**Keywords:** Biophysics, Biomaterials, Biomaterials - cells, Cell migration, Collective cell migration

## Abstract

Surface topography is a key parameter in regulating the morphology and behavior of single cells. At multicellular level, coordinated cell displacements drive many biological events such as embryonic morphogenesis. However, the effect of surface topography on collective migration of epithelium has not been studied in detail. Mastering the connection between surface features and collective cellular behaviour is highly important for novel approaches in tissue engineering and repair. Herein, we used photopatterned microtopographies on azobenzene-containing materials and showed that smooth topographical cues with proper period and orientation can efficiently orchestrate cell alignment in growing epithelium. Furthermore, the experimental system allowed us to investigate how the orientation of the topographical features can alter the speed of wound closure in vitro. Our findings indicate that the extracellular microenvironment topography coordinates their focal adhesion distribution and alignment. These topographic cues are able to guide the collective migration of multicellular systems, even when cell–cell junctions are disrupted.

## Introduction

In vivo, the majority of the cells reside inside or on top of the extracellular matrix (ECM), which is an environment rich in sugars, ions and often fibrous proteins such as collagen^1^. Cells intimately interact with each other and with the ECM via different biochemical and biophysical signals^2–4^. It is well established that the biophysical cues (e.g. tensile forces, environment stiffness and topography, shear flow) co-regulate cellular physiology, differentiation, proliferation, and migration^5–7^. In particular, the arrangement of surface features of the ECM (i.e. topography) serves as a template for cellular and tissue organization, and active remodelling^2^. These processes often require persistent cell migration over long distances in a collective manner. Collective migration, a process by which a group of cells moves in concert without completely disrupting their cell–cell contacts, is prominent during embryonic morphogenesis, but also in tissue repair and regeneration (e.g. wound healing)^8–10^. Enhanced migration and invasiveness characterize also pathological events, such as cancer metastasis, where malignant cells invade the surrounding tissue^11^. In this process, the orientation of the external constraints of the ECM is considered as an important guiding factor for cell migration^12–14^.

During decades of research, significant understanding has been achieved especially on single cell migration^9,15^. However, the used in vitro migration models are often limited in terms of complexity and therefore the role of biophysical properties of the ECM on multicellular processes is still in its infancy^8,16–20^. The effect of geometrical confinement of epithelial cell sheets on collective migration has been mainly studied by controlled deposition of adhesive proteins on the culture substrate. These studies suggest that confinement of cell sheet can alter the cell migration mode and speed^18,21–27^. However, to the best of our knowledge, the influence of topographical signals on collective migration of epithelial cells has not been studied in terms of the distribution of the main cell-ECM contact sites, focal adhesions^19,20,28–30^. The localizations of these adhesions are known to drive directed single cell migration^5,6^. However, in epithelium, dynamic cell–cell junctions and their proteins add an additional level of complexity to the regulation of collective migration. It is well established that cadherins and catenins of the adherens junctions, and zonula occludens proteins of tight junctions are important regulators of intercellular forces and epithelial migration^31^. However, it is not clear whether topographical signals influence the collectivity of cell movements similarly as it occurs in single cells^6,30^.

In this context, there is a need for developing novel stimuli-responsive materials for investigating the mechanobiology of multicellular systems^8^. Smart responsive biomaterials have been implemented as cell culture substrates and, in particular, the use of photosensitive materials has recently been gaining popularity^32^. Among these, azobenzene-based materials have the unique ability of modifying their surface microtopography in response to incident light. These materials, and especially photoinscribed sinusoidal surface relief gratings (SRGs)^33^ have been demonstrated to be a useful tool to induce single cell elongation and alignment in response to an underneath surface microtopography^34,35^.

Epithelial cell sheets have recently been modelled as ordered nematic liquid crystalline materials^36^. The nematic mesophase of liquid crystals comprises rod-like molecules that flow in a collective, ordered manner whose directionality can be controlled with external stimuli^37,38^. Seeking this new analogy, the influence of surface topography on collective cell movements can be anticipated, in the same way as surface features dictate the orientation of nematic liquid crystals^39,40^. Several research questions can be formulated based on this model. Can a surface topography direct and organize the migration of a multicellular layer in the same way as grooved substrate can orient single cells? What is the role of focal adhesions in the oriented collective migration process?

Herein, we use microtopographic photopatterns on a thin film of azobenzene molecular glass to address the questions above and to investigate the effect of smooth topographic features on collective migration of epithelial cells. We show that sinusoidal microtopography with 1 µm periodicity is particularly efficient in directing the epithelial cell migration and confined distribution of focal adhesions. Live-cell microscopy and immunolabelling experiments allowed us to unravel the effect of periodic surface microtopography on focal adhesion sites during collective epithelial migration and their impact on wound closure. Moreover, these results pave the way for application of azobenzene-based materials for the study of epithelium mechanobiology in dynamic conditions, applications in smart bandages and photoactive cell culturing platforms.

## Materials and methods

### DR1-glass sample preparation

Square glass coverslips were ultrasonicated twice in acetone for 10 min. The azobenzene-containing glass-forming material (Disperse Red 1 molecular glass, DR1-glass, Solaris Chem Inc., Fig. S1a) was dissolved in chloroform (5% w/v). The solution was dispensed over the cover glass (Laurell Technologies Corporation) at 1,500 rpm for 30 s, yielding uniform, high-quality thin film with thickness of ca. 400 nm. The films were photopatterned using Lloyd’s mirror interferometer^41^. The inscription was performed with a 488 nm continuous-wave laser (Coherent Genesis CX488-2000) using an intensity of 300 mW/cm^2^ over an area of 0.25 cm^2^. The polarization of the inscription beam used was horizontal or circular to induce efficient formation of SRG^42^. The microtopography period Λ was given by $\Lambda =\frac{\lambda }{2\mathrm{sin}\vartheta }$, where λ is the laser wavelength and ϑ is the angle between the laser beam and the mirror. By varying ϑ, sinusoidal microtopographies with different period can be produced. The inscription of the gratings was monitored with a low-power (1 mW) 633 nm He–Ne laser. The diffraction efficiency is defined as the ratio of the power of the first-order diffracted beam to the power of the beam transmitted through an unexposed spot on the sample. A JPK NanoWizard II (Bruker Nano GmbH, Berlin, DE) atomic force microscope (AFM), mounted on the stage of an Axio Observer Z1 microscope (Carl Zeiss Microscopy GmbH, Göttingen, DE), was used to characterize the microtopographies surface modulation. A silicon nitride tip (MLCT, Bruker Nano GmbH) with a spring constant of 0.01 N/m was used in contact mode in air at room temperature. Additional AFM studies on collagen-coated substrates were performed with Park XE-100 AFM (Park Systems, USA) in non-contact mode in air with an Al coated Si ACTA probe (AppNano, USA) nominal frequency 200–400 kHz, spring constant 13–77 N/m, as well as by Cypher ES, (Oxford Instruments Asylum Research, High Wycombe, UK) in tapping mode with blueDrive actuation with an Arrow UHF probe (NanoWorld AG, Switzerland), 700–2000 kHz nominal frequency.

### Cell culture

Epithelial Madin–Darby canine kidney type II (MDCK II) cells were cultured in MEM GlutaMax supplemented with fetal bovine serum (10%) and penicillin/streptomycin (1%). Cells were cultured at 37 °C in a humidified atmosphere with 5% CO_2_. Before cell seeding, DR1-glass substrates were sterilized under UV light for 30 min and coated either with a solution of monomeric rat tail type I collagen (50 μg/ml, Thermo Fischer Scientific, Waltham, Massachusetts, USA) in 0.02 N acetic acid for 30 min, or fibronectin (purified from human plasma, 50 µg/ml) in phosphate buffered saline (PBS).

### Immunolabelling and imaging

Cells were fixed with 4% paraformaldehyde for 15 min, treated with the permeabilisation buffer (0.5% BSA, 0.5% Triton-X 100, 0.1% NaN_3_ in PBS) for 15 min, and blocked for 1 h with 3% bovine serum albumin-PBS solution. Then, the samples were immunolabelled with mouse anti-vinculin (1:250, Sigma-Aldrich, Saint Louis, USA #V9131) and/or rabbit anti-pFAK (1:200, Abcam, Cambridge, UK, #ab81298). Secondary antibodies were anti-mouse-Alexa 568 (1:200, Thermo Fisher Scientific #A10037) and/or anti-rabbit-Alexa 647(1:200, Thermo Fisher Scientific, #A21245). Atto-550 or 565-phalloidin (1:50, Sigma-Aldrich #19083 or #94072) was used to label the actin cytoskeleton. Finally, samples were mounted with ProLong Gold antifade mountant with or without 4′,6-diamidino-2-phenylindole (DAPI) (Thermo-Fisher Scientific, #P36935) for staining cell nuclei. Confocal microscopy of the samples was conducted using Nikon A1R laser scanning confocal microscope mounted in inverted Nikon Ti–E (Nikon Instruments Europe BV, Amsterdam, Netherlands). The used excitation laser lines and emission filters were 405 nm, emission 450/50 nm; 561 nm, emission 595/50 nm; and 633 nm, emission 700/75 nm. The laser intensity was adjusted to minimize photobleaching. The images were collected using 60×/1.4 Plan-Apochromat oil immersion objective. The detector sensitivity was adjusted for each sample to optimize the image brightness and to avoid saturation. The images were 1,024 × 1,024 pixels with a pixel size of 114 nm in x/y. The data was collected as 3D z-stacks, each containing 20–30 slices with 250 nm interval.

### Wound healing experiments

In wound healing experiments, poly(dimethylsiloxane) (PDMS) slices (1:10 w/w, curing agent: Sylgard 184 pre-polymer, Sigma-Aldrich) about 0.3 × 1.5 cm in size, were cut with a sterile blade and positioned on the DR1-glass-coated substrates either orthogonally to the microtopographic pattern direction or alongside it. On flat samples the slices were placed in an arbitrary direction. Nikon BioStation CT was used to perform MDCK II migration experiments. PDMS slices were removed in a sterile environment and then the samples were kept 1 h in the Biostation incubator before starting the experiment, in order to allow temperature to equilibrate. The interval between images was 20 min for a maximum of 5–6 days without medium change.

### ZO-1/ZO-2 knock-out cell lines

ZO-1, ZO-2 double knockout cell line was generated by first creating a stably expressing-Cas9 cell line. A TJP1-targetting pLenti-gRNA-puro plasmid (gRNA sequence: GTAATTTCAGATGTGCTGAA) was then transduced into Cas9-expressing cells before selection for 6 days. Single knockout was confirmed with immunofluorescence imaging of ZO-1. Clonal lines were then created by dilution plating in a 96-well tissue culture dish. Knockout of clonal lines was verified with western blotting and genomic sequencing. A double knockout cell line was created by repeating the same protocol above with a TJP2-targeted gRNA (gRNA sequence: GTACACTGTGACCCTACAAAA). Double knockout of clonal lines was verified with western blotting against ZO-1 and ZO-2 and genomic sequencing^43^.

### Antibody blocking experiments

For the E-cadherin blocking experiment, Anti-E-Cadherin antibody [DECMA-1] (#ab11512) was filtered to eliminate sodium azide used as a preservative and diluted in PBS with a final concentration of ca. 500 µg/ml. In blocking, the working concentration was 5 µg/ml and the antibody was supplemented 1 h prior to PDMS stencil demoulding. Time lapse microscopy was performed with EVOS FL auto (Thermo Fisher Scientific, Massachusetts, USA). Four fields for each sample were imaged. Cells were fixed with 4% paraformaldehyde for 15 min, treated with the permeabilisation buffer (0.5% BSA, 0.5% Triton-X 100, 0.1% NaN_3_ in PBS) for 15 min, and blocked for 1 h with 3% bovine serum albumin-PBS solution. Then, the samples were immunolabelled with rabbit anti-pFAK (1:200, Abcam, Cambridge, UK, #ab81298). Secondary antibodies were anti-rabbit-Alexa 647 (1:200, Thermo Fisher Scientific, #A21245), anti-rat-568 (1:200, Thermo Fisher Scientific, #A-11077). Atto-488-phalloidin (1:50, Sigma-Aldrich, #49409-10NMOL) was used to label the actin cytoskeleton. Finally, samples were mounted with ProLong Gold antifade mountant with or without 4′,6-diamidino-2-phenylindole (DAPI) (Thermo-Fisher Scientific, #P36935) for staining cell nuclei.

Cell culture in low Ca^2+^ concentration was achieved by supplementing cell culture MEM GlutaMax medium with dialysed fetal bovine serum to reduce the Ca^2+^ concentration. Cells were washed twice by centrifugation before seeding. After 3 days of culture, cells were fixed and immunostained as described above.

### Quantitative analyses and statistics

Evaluation of cell orientation was performed using the OrientationJ plugin in ImageJ distribution Fiji software^44,45^. Orientation in degrees and morphological coherency were measured either locally, or for the entire field of view and reported as a function of time. Particle Image Velocimetry analysis was carried out using PIVlab MATLAB plugin on consecutive image pairs^46^. The analysis was performed using a 3-pass analysis with first pass interrogation window of 128 × 128 pixels with a 50% overlap. For wound healing analysis a 4-pass analysis with first pass interrogation window of 64 × 64 pixels (50% overlap) was used. A fast Fourier Transform (FFT) method was used for window deformation. Cell flow (magnitude and orientation) was extracted as frame average value, whereas the order parameter and the correlation distance were performed on MATLAB from smoothed vector fields. The order parameter S^47^ is defined as follows:

1 $$S = \left\langle {2\left( {\cos \vartheta } \right)^{2} - 1} \right\rangle$$

where ϑ is the angle between the pattern direction (always placed vertically in the images) and the velocity vector direction and it is averaged over each frame. S is 0 when there is no orientation, 1 when the vectors are oriented in the pattern direction, and − 1 when the vectors are orthogonal to the pattern direction. The final plotted data are the result of the average of all the available fields and smoothed by applying a moving average over 5 h. From velocity vector fields, a correlation coefficient^47^ was also calculated as follows:

2 $$Corr\left( {\overline{r},t} \right) = \frac{{u^{*} \left( {\overline{r}^{\prime } + \overline{r},t} \right) \times u^{*} \left( {\overline{r}^{\prime } ,t} \right)_{{\overline{r}^{\prime } }} }}{{\left[ {u^{*} \left( {\overline{r}^{\prime } ,t} \right)^{2} u^{*} \left( {\overline{r}^{\prime } + \overline{r},t} \right)^{2} } \right]^{1/2} }}$$

where u (or v) are the horizontal and vertical components of the velocity vector and $\stackrel{-}{u}$ (and $\stackrel{-}{v}$) are the mean velocities over the frame. The curves have been calculated with respect to one point few lines behind the wound edge. The graphs in Fig. 6 represent the average of 3 h for each of the four time points of interest (0 h, 24 h, 48 h, and 96 h). The final plots are reported as moving average over a distance of 5 pixels. The ImageJ plugin FFT was used for quantifying the vinculin distribution through the measurement of a 2D Fourier transform of both the vinculin channel for focal adhesions and the transmitted light channel for the SRG at the cell-material interface. The quantification was performed by partly following the protocol introduced by Taylor et al.^48^. Briefly, different circular regions were masked from microscope images in Corel Photo-Paint X7 and FFT was then performed on ImageJ. Later, the periodicity was evaluated by comparing the peak-to-peak distance (termed as length) between the zero and first order diffraction maxima of the vinculin and microtopographic pattern with the following equation:

3 $$Periodicity = \left( {1 - \frac{{\left| {length_{vinculin} - length_{pattern} } \right|}}{{length_{pattern} }}} \right) \times 100\% .$$

The alignment was derived by comparing the orientations of the first order diffraction maxima (angle) in the frequency space of the vinculin and microtopographic pattern channels as follows:

4 $$Alignment = \left( {1 - \frac{{\left| {angle_{vinculin} - angle_{pattern} } \right|}}{{angle_{pattern} }}} \right) \times 100\% .$$

Information on focal adhesion orientation and aspect ratio was obtained by pFAK immunostaining. Focal adhesions were analysed with the MomentMacroJ version 1.3 script (hopkinsmedicine.org/fae/mmacro.htm). The principal moments of inertia (i.e. maximum and minimum) were extracted and the aspect ratio was calculated, defined as the ratio of the principal moments; high values of aspect ratio identify elongated focal adhesions. Focal adhesion orientation was defined as the angle between the maximum axis and the pattern direction.

In the case of cell migration on different microtopographies, the images were collected from four positions in the case of patterned samples (0.5 µm, 1 µm pattern, 2.8 µm), and two positions in the case of flat control sample. For collective migration experiments, the data was imaged from six positions of parallel orientation, nine positions of orthogonal orientation and six positions of flat substrate. The experiment was conducted twice. The focal adhesion aspect ratio and orientation were quantified from randomly selected fields (the imaged field was selected based on DAPI staining), the goal was to have at least 30 quantified focal adhesions per condition. Sample sizes in the statistical analysis of focal adhesion aspect ratio were n = 67–84 for orthogonal wound orientation, (0 h n = 67, 24 h n = 84, 48 h n = 82, 96 h n = 80), n = 43–87 for parallel wound orientation, (0 h n = 50, 24 h n = 87, 48 h n = 74, 96 h n = 43) and n = 52–105 for flat substrate (0 h n = 72, 24 h n = 71, 48 h n = 105, 96 h n = 52). Sample sizes in the statistical analysis of focal adhesion orientation were n = 67–73 for orthogonal wound orientation (0 h n = 66, 24 h n = 73, 48 h n = 65, 96 h n = 73), n = 42–87 for parallel wound orientation (0 h n = 48, 24 h n = 87, 48 h n = 74, 96 h n = 42) and n = 35–98 for flat substrate (0 h n = 69, 24 h n = 78, 48 h n = 98, 96 h n = 35). Only data with non-optimal technical quality (e.g. focus drift during imaging) was excluded from the analysis. Statistical analyses were performed in MATLAB. Our data were found to have non-normal distributions, therefore the nonparametric Kruskal–Wallis test with Dunn–Sidak post-hoc analysis was used to assess statistical significance.

## Results

### Generation and characterization of collagen-I coated DR1-glass sinusoidal surface topography

**Figure 1:**
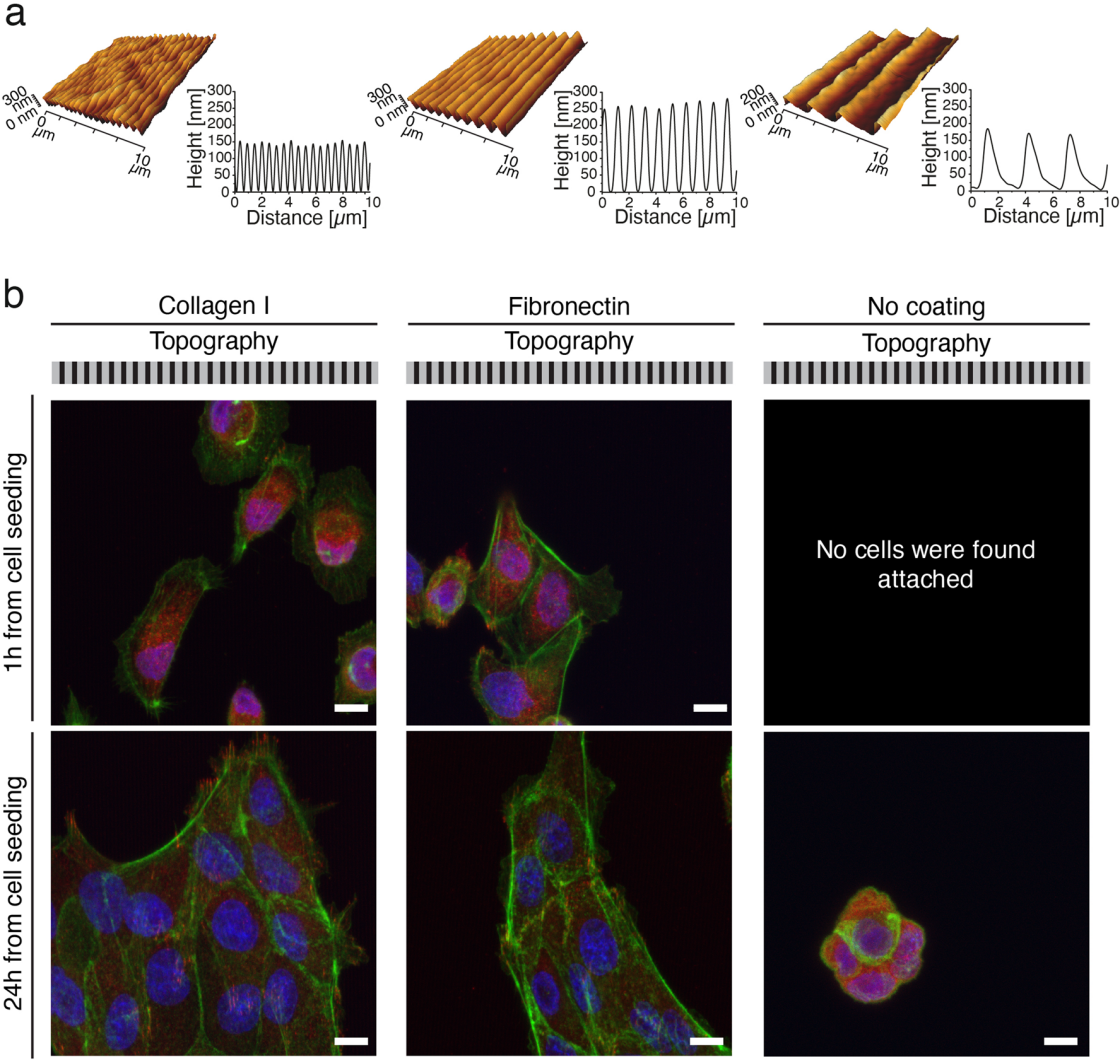
Substrate microtopography properties and cell adhesion with different coatings. **a** AFM 3D renderings and surface profiles of the inscribed topographies (from left to right: 0.5 μm, 1.0 μm and 2.8 μm). **b** Immunolabelling of MDCK cells on different coatings at different cell seeding time points. Actin (phalloidin stain, green) was labelled together with pFAK (red) and chromatin (DAPI stain, blue). The sketch of the topography illustrates the presence of a microtopography. Scale bars are 10 μm.

Azobenzene-containing amorphous thin films can be topographically patterned using light stimulus inducing cyclic and reversible photoisomerization of the azobenzenes^41^. Such surface microtopographies have recently been successfully implemented in single cell studies, highlighting the remarkable potential of azobenzene-containing materials in devising stimuli-responsive biointerfaces^35,49^. We used a glass-forming DR1-containing material (DR1-glass, Fig. S1a) as novel material for epithelial cell culture^50^. DR1-glass yields efficient and reproducible surface deformation upon irradiation with blue light interference pattern due to its monodispersity (see UV/Vis absorption spectrum in the solid state in Fig. S1b)^51^. We generated large-area (area size range $\sim$ cm^2^), sinusoidal surface features by exposing the substrates for 10 min to 488 nm interference pattern, while monitoring the formation of the topography via in situ diffraction measurements at 633 nm (Fig. S1c). The periodicity of the surface structures was set to 0.5 μm, 1 μm and 2.8 μm, as determined by the angle between the two interfering laser beams. The surface microtopographies were characterized with an atomic force microscope (AFM), revealing surface modulation depth between 150 and 250 nm with the expected periodicity (Fig. 1a). Different surface coatings by ECM proteins were tested by using monomeric type I collagen, fibronectin, and uncoated substrates (see “Materials and methods” section). MDCK II cells adhered promptly to the type I collagen- and fibronectin-coated DR1-glass surfaces, forming a confluent epithelium after approximately 2.5 days after seeding (Fig. S2), whereas their adhesion onto uncoated substrates was slower (Fig. 1b). We decided to use collagen I for all the following experiments since, when deposited on the substrates in its monomeric form under acidic conditions, it provides cell adhesive ligands of collagen I without masking the presence of the pattern via formation of fibres (Fig. S3). Furthermore, during collective migration in wound healing, especially in the epithelialization phase, epithelial cells migrate on collagen rich ECM^52^. AFM analysis on the collagen-coated substrates was performed under different coating conditions, revealing that no fibres formed over the substrate in the working concentration (Fig. S3), supporting the use of collagen I in our studies.

### Topography-guided collective behaviour and focal adhesion confinement

**Figure 2:**
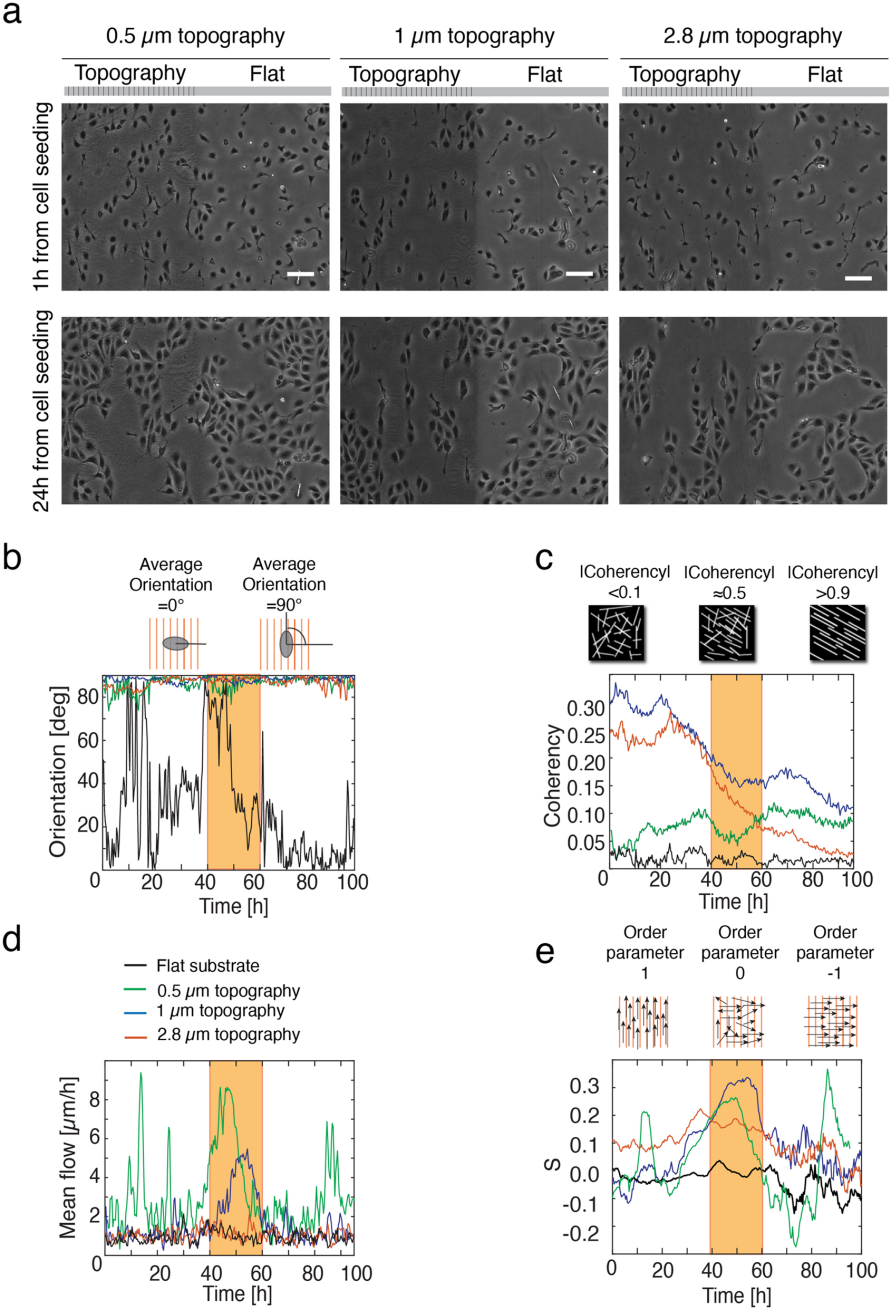
MDCK II cell colony growth and migration on different topographies. **a** Phase contrast images of MDCK cells during colony growth on surfaces with different topographic features. The images were taken 1 h (top) and 24 h (bottom) after seeding. Scale bars are 100 μm. The sketch illustrates the division of the field to microtopographic and flat regions. **b**–**e**, Progression of orientation, coherency, mean flow magnitude, and order parameter, during cell colony growth and migration.

In order to decipher whether the sinusoidal surface patterning could drive epithelial collective behaviour, we analysed the cellular orientation and morphological coherency. When seeded on different microtopographies (Fig. 2a), MDCK II cells rapidly aligned along the topography direction already 1 h post-seeding. After 24 h, the cells were typically arranged in small colonies, whose extent of elongation depended on the topography period (Fig. 2a, Supplementary movie 1). The alignment of the cells on the patterned substrates was quantified throughout their growth in terms of the morphological parameters of orientation and coherency from phase contrast images^53,54^. The orientation parameter is defined as the angle of dominant direction of the image features with respect to the horizontal axis (Fig. 2b). Coherency represents the morphological anisotropy in the image features averaged in a defined region of interest (Fig. 2c): coherency = 0 means that the features are isotropic, while coherency > 0 indicates a dominant direction in the features shape. When averaging over the entire field of view, a larger coherency represents more elongated and aligned features. In Fig. S4, the corresponding local coherency and orientation are shown. On the flat substrates, the average orientation of the cells was random, whereas, on the microtopographies, the cells oriented themselves along the topography ridges (Fig. 2a–c). The quantification indicated that the coherency reduced during epithelium formation (Fig. 2c). However, on the 1 μm and 2.8 µm patterns, the cells followed the surface microtopography more efficiently for a longer time when compared to the 0.5 µm pattern and the flat substrate (Fig. 2c). The results show that microtopography direction and its periodicity induce uniform cell orientation during cell proliferation and growth.

Next, the effect of surface microtopography on cell colony orientation and migration speed was analysed by particle image velocimetry (PIV), which indicated an increase in the mean cellular speed especially between 40 and 60 h post seeding (represented with an orange stripe in the graphs) on 0.5 µm and 1.0 µm topography patterns, but not on the 2.8 µm patterns or on flat substrate (Fig. 2d and Supplementary movie 2). The 40–60 h phase corresponds to the final closure of gaps between the cells with the consequent formation of a confluent epithelium. From PIV data, we also calculated the order parameter S (Eq. 1 in “Materials and methods”), which quantifies the overall orientation of velocity fields and gives a good indication of the collective displacements of the cells (Fig. 2e). Thus, as coherency and orientation are measure of feature morphology in terms of elongation and orientation, order parameter S describes the collective orientation of the velocity vector field. In accordance with the previous analyses, the cells on the flat films did not show any coordinated displacement, while those on the 1 µm pattern displayed increased collective orientation.

Taken together, these results support our view on topography-driven collective behaviour of epithelial cells. The topographical signals only from the 1 µm pattern led into a strong response in the cell behaviour in all four parameters of interest: orientation, coherency, cellular flow rate, and order parameter. Interestingly, the size and shape of these surface features correlate with the in vivo dimensions of type I collagen fibres^55^. Therefore, the topographic pattern with 1 µm period with collagen coating was selected for the experiments that follow.

### Effect of topographical cues on focal adhesion orientation during epithelium formation

**Figure 3:**
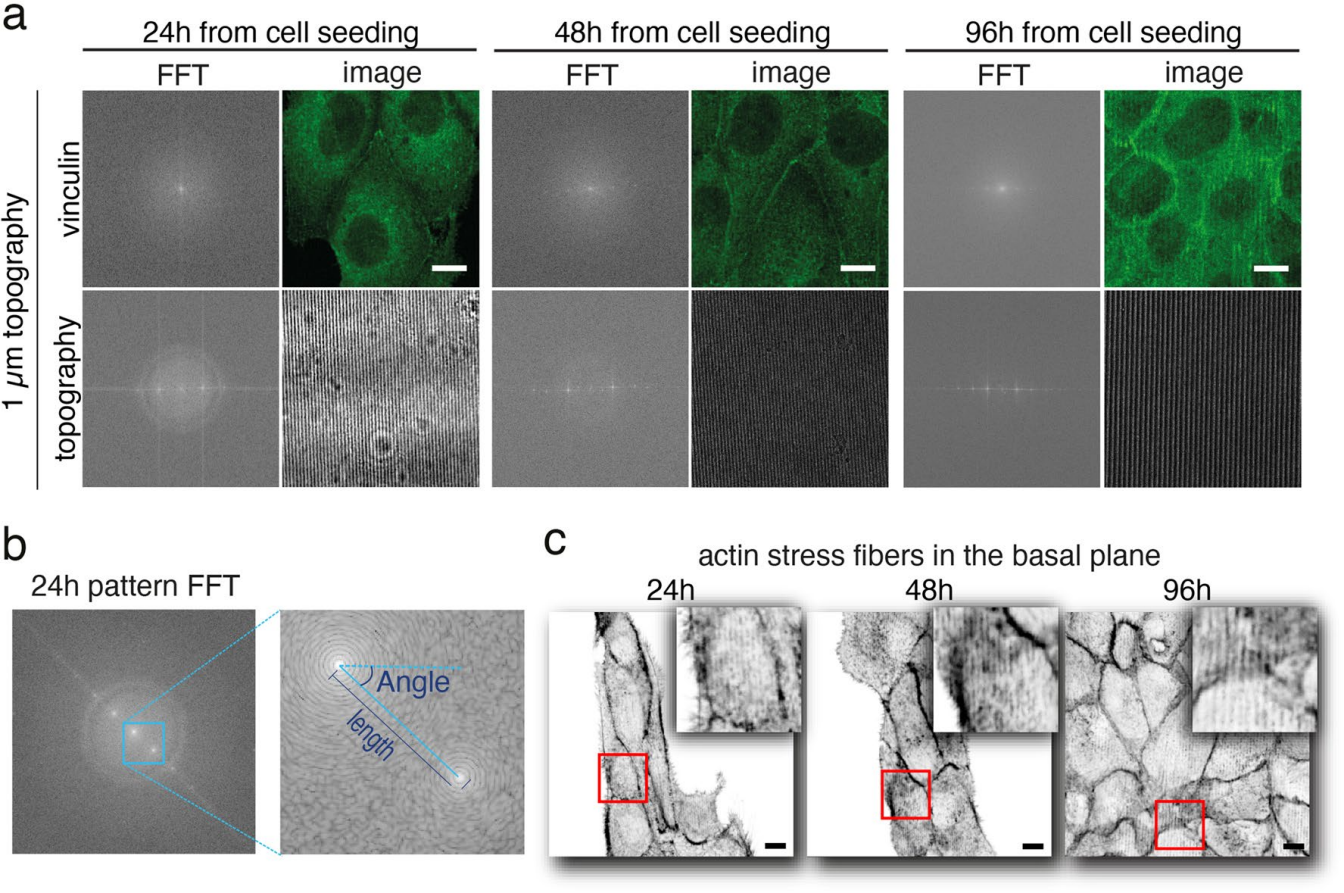
Analysis of the organization of focal adhesions and actin cytoskeleton. **a** The analysis on the focal adhesion organization on the substrates was performed by using fast Fourier transform (FFT) of the vinculin image and the brightfield image of the underneath topography. Scale bars 5μm. **b** Quantification of the focal adhesion orientation and periodicity was performed by measuring and comparing the length and the angle formed with the horizontal axis of the first order diffraction signal of the two channels (see text for the related equations (3) and (4)). **c** Actin stress fibres at the basal plane as defined by phalloidin staining. Scale bars 5μm.

The previous experiment suggested that surface microtopography can effectively induce collective alignment and migration of epithelial cells during cell colony growth. At the single cell level, the topography of the cell-ECM interface, together with cellular contractility, co-regulates cell behaviour through focal adhesions by presenting discontinuous zones (e.g. ridges or posts) that favour patterned and oriented integrin clustering and influence cell shape, signalling, membrane dynamics and migration^27,56–58^. Thus, we hypothesised that the cellular contractility and membrane deformability could define the limits for surface microtopography sensing also in multicellular systems. To this end, we investigated the effect of topographical cues on focal adhesion orientation during epithelium formation. This was achieved by immunolabelling vinculin, one of the main components of mature focal adhesions^59^, at different time points post-seeding (24 h, 48 h, and 96 h) (Fig. 3). The vinculin distribution was evaluated by comparing the periodicity of the vinculin signal and brightfield transmitted light signal of the topography. The image data was converted into frequency space using fast Fourier transform (FFT). The FFT analysis revealed that on the microtopographically patterned substrates, vinculin shared the same periodic organization as the underneath topography (percentual periodicity and alignment between 99 and 100%) (Fig. 3a,b). On flat substrates, instead, no first order peaks in spatial frequencies in the vinculin signal were detected (Fig. S5), indicating non-periodic distribution of focal adhesions. Moreover, basal actin stress fibres, as identified by phalloidin staining, were found locally oriented in the topography direction and periodically organized (Fig. 3c). As a control, signal from DAPI-labelled chromatin from the nucleus was used to ensure that the observed periodicity did not arise from optical aberrations (Fig. S6). Together, these results suggest that sinusoidal topography induces confinement of focal adhesions on the pattern ridges during the growth of the epithelial monolayers.

In order to understand the mechanisms of the collective cell alignment on the patterned surfaces, we used pharmacological inhibitors to block specific signalling pathways involved in the regulation of actin cytoskeleton. For that, two parallel signalling pathways were blocked by inhibiting RhoA-dependent kinase (ROCK) or phosphatidylinositol-3-kinase (PI(3)K), both related to cell-ECM interactions and responsible for balancing cellular contractility and cell membrane protrusion formation respectively (see Supplementary Information for experimental details)^16,60^. These experiments showed that inhibition of the PI(3)K increased whereas ROCK inhibition decreased collective cell alignment on the microtopographies (Fig. S7 and Supplementary movie 3). The results suggest that the activity of the ROCK pathway, and thus active cell contractility, is required for the efficient cell orientation and migration. All in all, it seems likely that cellular stiffness and the interplay between focal adhesions and basal actin stress fibres contribute to the collective cell alignment as a response to topographical cues.

### Effect of topographical cues on wound healing

**Figure 4:**
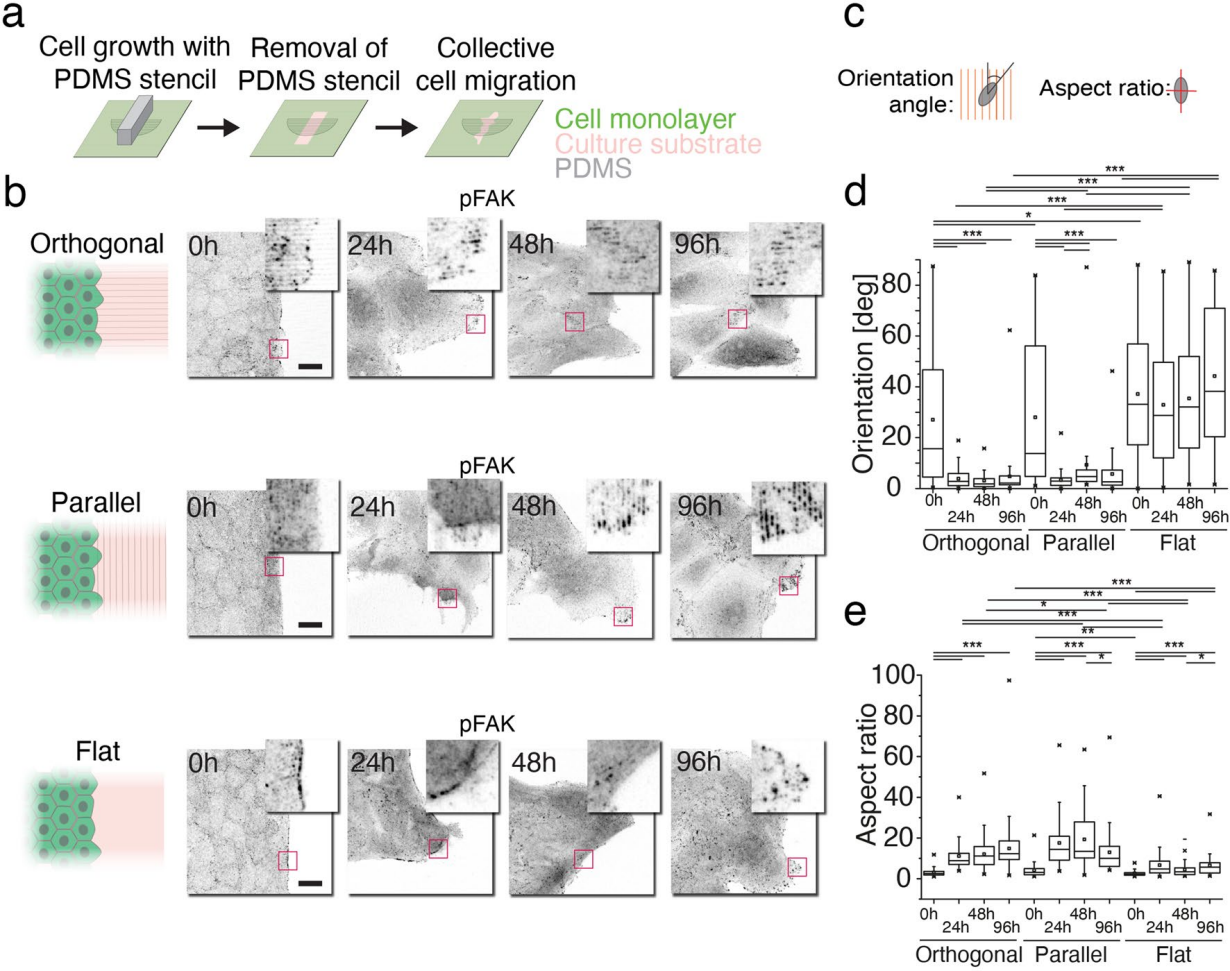
Analysis of focal adhesion orientation and shape during wound healing. **a** Schematic representation of wound healing experiment. **b** Orientation of the wound in comparison to 1 μm topographical features and the pFAK immunolabelling (inverted grey scale) of migrating wound edge at different time points in orthogonal, parallel and flat configurations. Scale bars are 20 μm. **c** Graphical representation of orientation angle and aspect ratio parameters. **d** and **e**, Orientation angle and aspect ratio at different time points in orthogonal, parallel and flat configurations. The numbers of the analysed events per group are reported in the Methods section. *0,01

To investigate how sinusoidal surface microtopography influences the dynamics of collective cell migration, in vitro wound healing experiments were performed (Fig. 4a)^27^. After removing the stencils, the epithelium was able to collectively migrate to the newly exposed surface containing either the 1 µm microtopographic pattern, or a flat surface, both previously coated with collagen-I. This experiment allowed studying how the topography affected collective cell movements during spontaneous migration towards the wound closure. We used three experimental configurations (Fig. 4b): one with the PDMS stencil placed orthogonally to the grooves of the pattern (orthogonal sample), one with the stencil placed along the pattern grooves (parallel sample) and a control sample with the stencil on top of a flat substrate (flat sample). In this experiment, focal adhesions were detected with immunolabelling of pFAK, of which autophosphorylation at tyrosine-397 residue indicates that focal adhesions are in their active state, it is a trigger to intracellular mechanotransduction pathways, and it is also associated with directed migration^59^. In single endothelial cells, pFAK activation has been reported to be more than 80% higher in cells adhering for 20 min to similar topography as our 1 µm pattern, when compared to flat control samples^58^. Analysing the localization and aspect ratio of pFAK signals allowed us to get a more precise mapping of nascent and topography oriented focal adhesions. As expected, pFAK localized strongly to the focal adhesions (Fig. 4b and Fig. S8) at different time points of wound healing (0 h, 24 h, 48 h, and 96 h). Both the orthogonal and the parallel samples shared similar cell dynamics, and focal adhesions progressively assembled along the topography direction, acquiring also an elongated shape (orientation and aspect ratio, Fig. 4c–e). In comparison, the flat sample presented a random orientation of roundish focal adhesions (Fig. 4d,e). In the orthogonal sample, the direction of assembled focal adhesions corresponded to the direction of wound closure, whereas in the parallel configuration, focal adhesions oriented just along the wound edge, most likely interfering with the closure. In conclusion, the results showed increased assembly, uniform orientation and elongation of focal adhesions along the patterns during formation of confluent epithelium and in wound healing, often accompanied by the local formation of basal stress fibres oriented along the pattern. These findings suggest that topographical guidance affects the wound healing capabilities of the epithelium.

**Figure 5:**
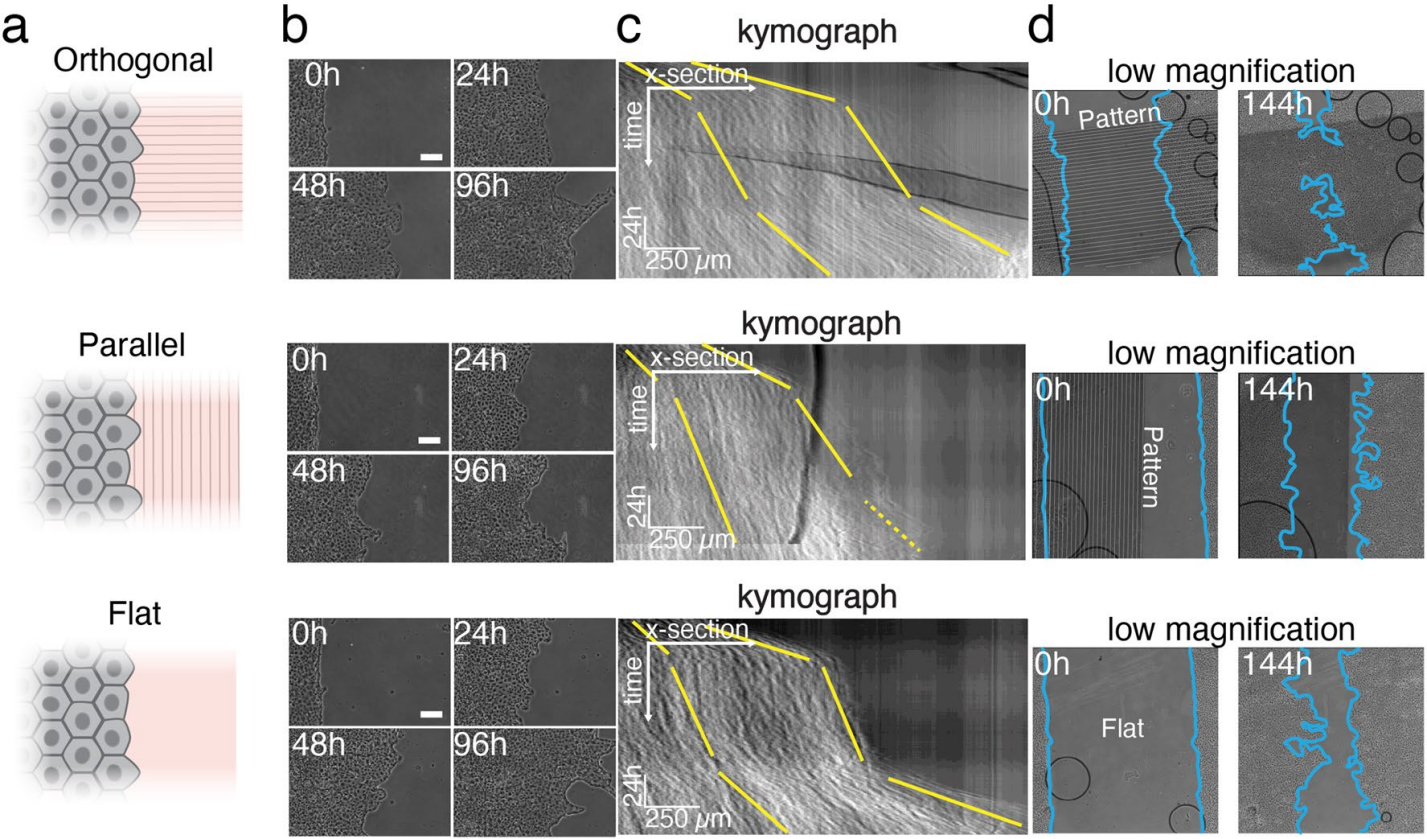
Migration of the epithelium in wound closure experiment. **a** Orientation of the wound in comparison to 1 μm topographical features. **b** Phase contrast images of the wound boundary progression over time. Scale bar 100 μm. **c** Kymographs of the wound closure (averaged over the entire field) from the phase contrast images. Yellow lines represent the progression of the front and the rear end of the epithelium (defined as 300 μm deep into the wound at the beginning of the process). The wound front in the parallel configuration during the third phase is indicated by a yellow dotted line due to its difficult identification. **d** Low magnification phase contrast images of the in vitro wound healing during migration on the three samples (flat, orthogonal, and parallel) right after PDMS slice has been peeled off and after 144 h (6 d). Light blue lines define the wound edges.

**Figure 6:**
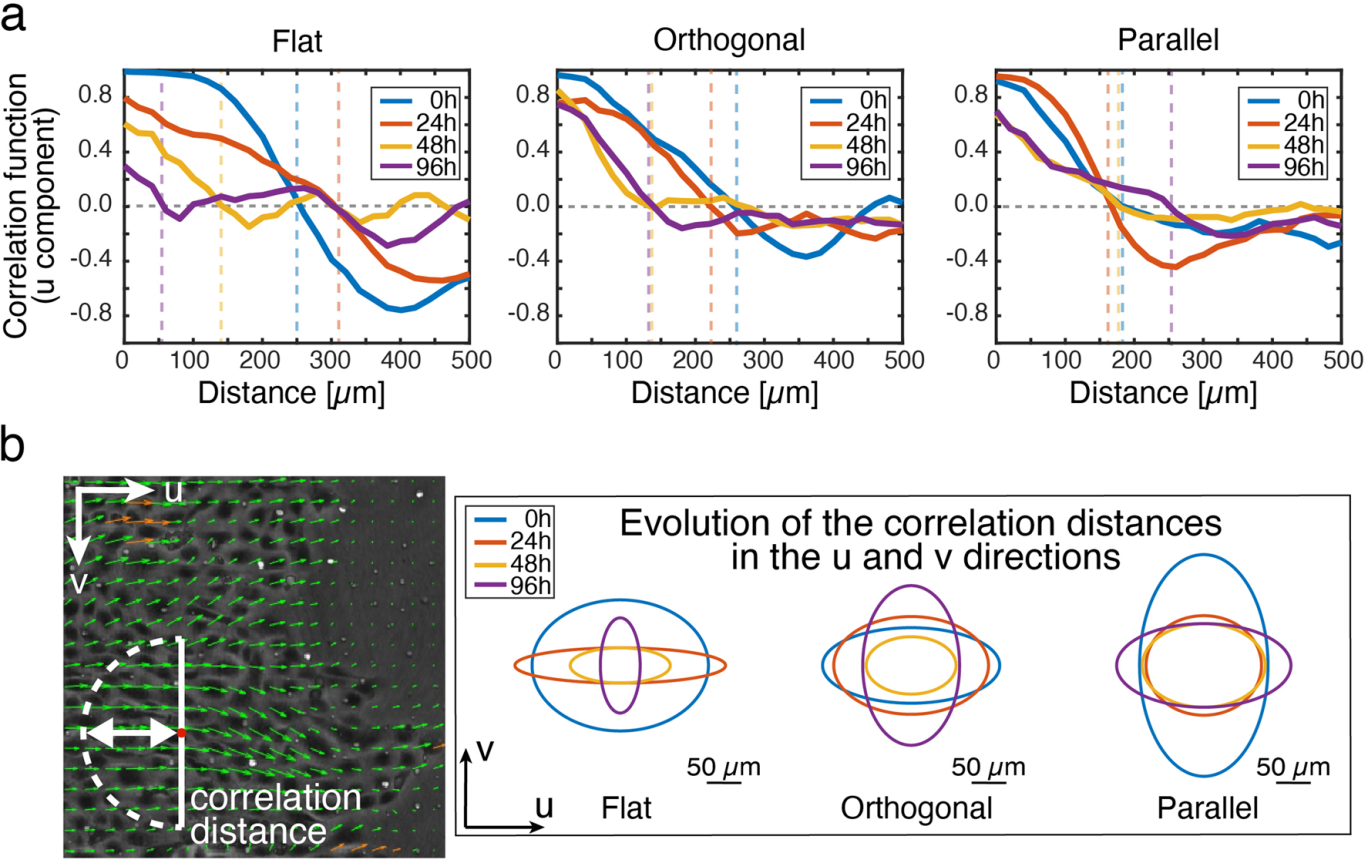
Collectivity of the epithelium migration in wound closure experiment on microtopographies. **a** Correlation function of the velocity vector u component (x direction, towards wound closure) at different time points. Each curve is mediated over 3 h and smoothed over a moving window of 5 pixels. The dashed lines highlight the positive correlation distance at each time point. **b** Illustrative sketch of correlation distance and time evolution of suchcorrelation distances of both u and v components in different pattern configurations. A higher correlation of the v component in the parallel sample can be identified.

Our results indicated that the collective migration of the epithelial cells was enhanced or slowed down, depending on the orientation of the 1 µm topographical features. In order to study this in more detail, time-lapse microscopy was conducted for six days to follow the dynamics of the epithelial gap closure in orthogonally or parallel wounded epithelium, with respect to microtopographic pattern configurations (Fig. 5a, Supplementary movie 4). To analyse the migration dynamics, kymographs from time lapse images representing the motion of the epithelium were reconstructed (Fig. 5b). These analyses indicated three distinct stages of the collective cell migration both on the flat sample and on the patterned substrate with the orthogonal wound orientation (Fig. 5c), while in the parallel wound configuration a proper identification of the third stage was difficult (yellow dashed line in the kymograph in Fig. 5c). The first stage, referred to as unjamming phase, corresponded to the fast advancement of the epithelium boundary with a duration of around 15 h^61^. By comparing the slopes of the leading and rear edges in kymographs during this first stage, a stretching of the leading edge was observed especially in the orthogonally wounded sample (Fig. 5c). The second stage (with a duration of 53 h) was characterized by the progression of the cell monolayer edge with a slower velocity than in the first stage, and the consequent jamming of the monolayer. Wound closure in the parallel sample experienced a sudden decrease in speed, whereas a smoother velocity profile was observed for the cells in the orthogonal wound sample, as additionally indicated by the kymographs. Interestingly, in the parallel sample the wound rear proceeded approximately four times slower than the border. In the third stage (with a duration of 28 h), the fast movement of the monolayer was again detected towards the final wound closure. Here, the different orientation of the microtopographies on the surface again substantially influenced the collective cell migration velocity. In particular, the wound closure in the orthogonally wounded sample proceeded almost twice as fast as in the parallel sample involving also the movement of cells in the back of the wound. After 6 days, the cells on the orthogonally wounded sample closed the wound region on the patterned area, whereas on the outside of the patterned area the wound fronts were clearly separated (Fig. 5d). Additionally, PIV analysis of the wound healing time lapse imaging were performed and the spatial correlation function of the velocity components along ($Corr u$) and across ($Corr v$) the wound closure direction (x direction) was calculated (Eq. 2). These factors define how dependent the cell velocities are and how far the displacement of one cell can affect the displacement of another. In Fig. 6a, the spatial correlation function of the u component is reported at the time points of interest (0 h, 24 h, 48 h, and 96 h) averaged over 3 h. From these graphs, a correlation distance was defined as the distance at which the correlation reaches zero value and the velocities are independent. First, we could affirm that the presence of the topography does not impair collective displacements of the cells and the correlation distance is in accordance with values reported elsewhere in the literature for the same cell line^47^. We could further observe differences in the correlation distances in presence of the microtopography. By comparing $Corr u$ and $Corr v$, we could see that the v component (y direction, along the wound edge and orthogonal to the wound closure direction) of the velocity is also correlated (Fig. 6b and Fig. S9). In the case of flat substrate, as one would expect, the velocity was more correlated in the direction of the wound closure and this anisotropy was reduced in time. When the topography was in the parallel configuration, instead, cell velocities were more correlated along the wound border than in the closure direction and this effect was reduced during migration. Conversely, when the microtopography was oriented orthogonally to the wound border, the correlation distance was bigger in the wound closure direction. Here as well, the anisotropy slowly decreased during the wound closure. To conclude, the wound healing experiment showed that in exactly the same cell density and culture conditions, the favourable orientation of the topography enhanced the healing of the wounded epithelium by promoting faster, collective displacements, whereas the closure in the parallel configuration was interfered by the presence of an unfavourable topography.

### Influence of cell–cell junctions in topography-aided cell migration

Finally, in addition to cues from the ECM, collective migration is strongly influenced by the cell–cell connections and interactions. Epithelial cells attach to the underlying ECM from their basal side and generate traction force. The apical cell–cell junctions transmit the force to neighbouring cells, leading to collective cell movements^8,62^. The important cell–cell junctions mechanically coupling the cells together include the tight junctions (TJs), which include the adaptor proteins ZO-1/ZO-2, and cadherin-based adhesions called adherens junctions (AJs)^63^. First, we studied the role of cell–cell junctions in cell colony and focal adhesion alignment by culturing wild-type (WT) MDCK cells in calcium depleted medium, compromising their inter-cellular interactions (Fig. S10). Besides a decreased monolayer growth speed, elongated cell islands in the pattern direction and confined focal adhesions were observed, suggesting that weakened cell–cell contacts via inhibited formation of E-cadherin do not impair the cell’s ability to follow the microtopographical guidance.

In order to investigate the role of cell–cell junctions in the ECM topography-guided collective cell migration, we used a cell line with a genetic knockout of ZO-1/ZO-2, impairing the TJs. First, we analysed the AJs, actin cytoskeleton and focal adhesion organization of the ZO-1/ZO-2 KO cells and compared that to the WT cells (Fig. S11). While the overall growth of the ZO-1/ZO-2 KOs was slowed down on the DR1-glas migrotopographies with respect to the wild-type cells, they grew into full confluency after 10 days. The focal adhesions were not so pronounced in pFAK labelling in ZO-1/ZO-2 KO cells as in WT cells but the overall organization of actin cytoskeleton was similar.

**Figure 7:**
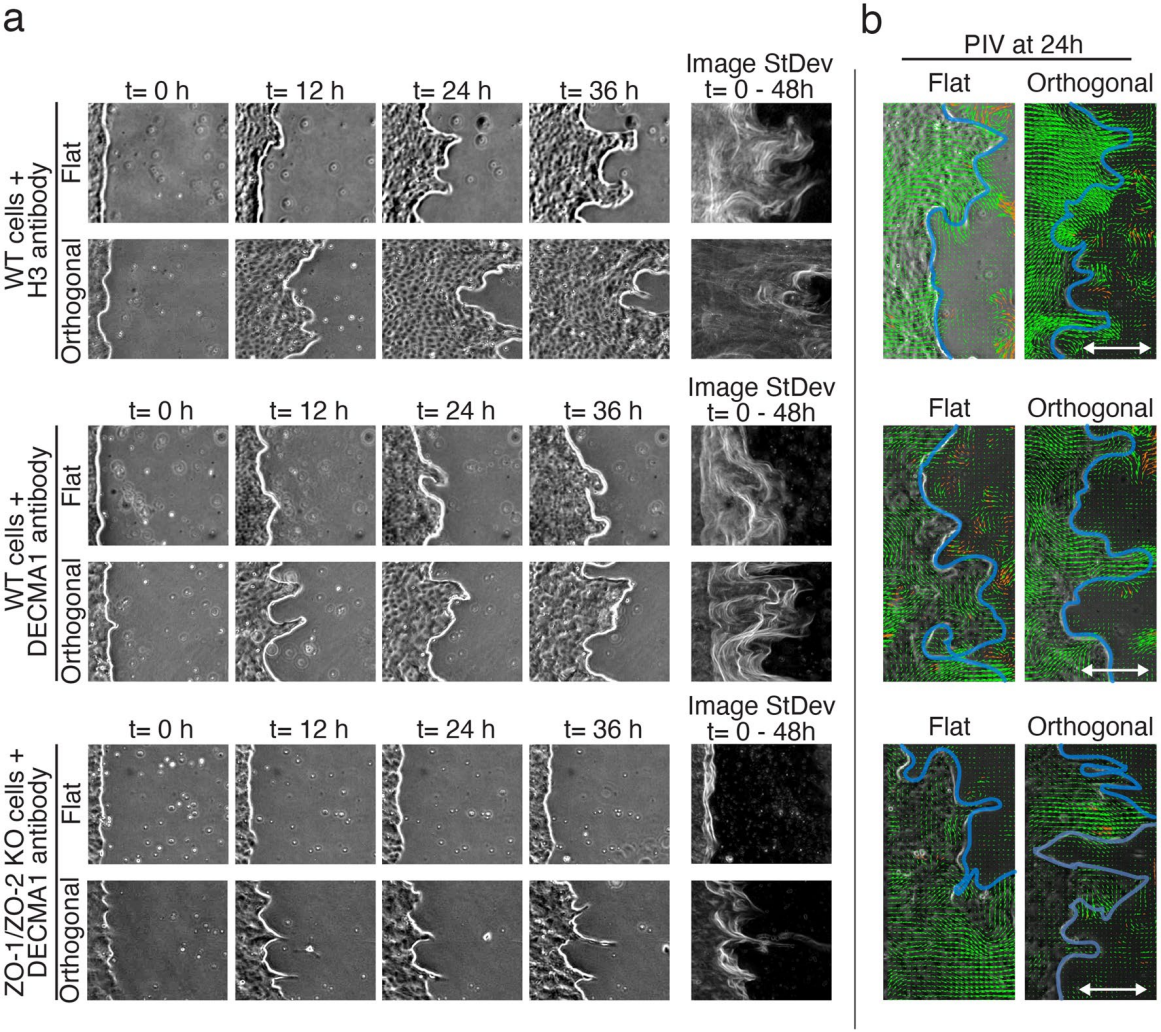
Influence of cell-cell junctions in the topography-aided cell migration. **a** Phase contrast images of the advancing wound edge at different time points (0h, 12h, 24h, 36h) in WT MDCK cells + H3 antibody (control), WT MDCK cells + E-cadherin blocking antibody DECMA1, and ZO-1/ZO-2 KO MDCK cells + E-cadherin blocking antibody DECMA1 both on flat substrates, as well as on orthogonal microtopography. On the right side, standard deviation projection of all the collected images showing the advancing fronts during 0 h - 48 h of wound healing. **b** PIV analysis of the collective migration of the MDCK cells at 24 h time point on flat substrate and orthogonal topography.

Furthermore, we disrupted the AJs by using an antibody (DECMA1) that blocks E-cadherin adhesion in WT and ZO-1/ZO-2 KO cells during a wound healing experiment. The blocking antibody localized to the cell–cell junctions in WT and especially in ZO-1/ZO-2 KO cells (Fig. S11). Next, we investigated the effect of cell–cell junction blocking on collective migration in wound healing. We used WT cells together with control antibody against histone H3, and WT and KO cells with E-cadherin blocking antibody. We let the cells migrate either on flat substrate or along the sinusoidal pattern (orthogonal orientation of the pattern). Disruption of adherens junctions by the blocking antibody reduced the advancement speed of the cell front, but in all cases the orthogonal pattern increased the migration (Fig. 7a,b). This was especially evident in the case of ZO-1/ZO-2 KO cells, on flat substrate the cell front stayed almost still for the whole 48 h imaging period, but the patterned substrate partially rescued the phenotype and induced collective migration of the cells (Fig. 7a). Furthermore, noticeable difference in formation of thin finger-like protrusions were observed with the ZO-1/ZO-2 KO cells combined with AJ disruption. Single cells were able to escape the leading front from the end of the finger-like structures on orthogonal topography, indicating strong guidance effect of the substrate (Supplementary movie 5).

In all, these experiments showed that the collective movement and cooperation of cells driven by focal adhesion alignment was acquired irrespective of cell–cell contacts and intercellular interaction, highlighting the importance of extracellular microenvironment topography guidance on collective behaviour of multicellular systems via focal adhesions.

## Discussion

In the epithelium, the communication and concerted behaviour of the cells is essential for many biological functions and tissue physiology^8^. These phenomena are related to the ability of cells to sense changes in their microenvironment (e.g. surface topography) and to alter their behaviour, including migration^9^. Collective cell migration drives essential multicellular organization and morphogenesis, and is important in pathological processes such as wound healing and cancer cell invasion. For instance, increased wound closure efficiency on topographical features has been reported in fibroblast studies in vitro. These studies have demonstrated an enhanced non-collective cellular migration by using topographical guidance from cell culture substrate (electrospun poly-ε-caprolactone) or apically positioned grooved PDMS^19,64^. Moreover, other studies have specifically focused on epithelial monolayers and tissue sheets using PDMS substrates with cell-adhesive or topographical patterns, polyurethane, silk, or polystyrene substrates with different topographical sharp and high-aspect-ratio features, showing straighter trajectories and aligned actin stress fibres in comparison to cells on a flat substrate^29,65,66^. Human retinal pigment epithelium derived cells (ARPE-19) have been cultured on a sinusoidal topography with 1 µm pitch made of PDMS^30^. Interestingly, in this study, the collective migration showed a significantly higher velocity and persistency compared to flat substrate irrespective of cell–cell junctions strength, in accordance with our results. The authors investigated the effect of contact guidance in dense cell sheets proposing a volume exclusion-type mechanism. However, in these as well as in other studies, the mechanistic understanding of contact guidance of the migration at the subcellular level in epithelium has remained elusive. This has been partly due to the lack of appropriate platforms that mimic the key physical features of the native ECM without major technical complexity.

In this study, we implemented DR1 molecular glass as novel photosensitive azobenzene-based biocompatible cell culture substrate, enabling the creation of large-area sinusoidal patterns with a tunable periodicity in a reproducible way. We showed that microscale topography leads to strong contact guidance of the epithelium, implying that the pattern orientation concomitantly drives focal adhesion confinement, basal stress fibres’ orientation, and the collective cellular motion. Currently, the exact role of topography on collective processes of epithelial cells is still under discussion. For example, it seems that the curvature of cell monolayer edge regulates the modes of collective cell motion (e.g. cell crawling in positively curved edges and supracellular contracting structures in negatively curved borders)^8,25,27^, but the influence of a non-homogeneous substrate has not been investigated. From our results, it seems that cell crawling mechanisms are associated with the topographical guidance, suggesting that topography-aided migration should be considered in shaping the next-generation epithelial mechanobiology models.

We attribute the effect of topography on the collective cell migration to the confinement of focal adhesions. Cell interaction with structured materials via focal adhesions and the consequent orientation and faster migration are all concepts that are well accepted and demonstrated for single cells. To date, this phenomenon has not, to the best of our knowledge, been investigated in epithelium. In our system, the focal adhesions progressively oriented and elongated along the grating grooves, and this phenomenon was coupled to the natural tendency of epithelium to unjam and close the wound, independently of physical cell–cell interaction and cooperation. Our observations agree with wound closure experiments in non-collective systems (e.g. fibroblasts)^64^ as well as in other collective systems like endothelium^6,58,67^. The extension of the contact guidance concept to multicellular systems, even if possibly inferred, was not obvious.

Our results are consistent with related studies done in vivo and in vitro. In vivo, during early-stage wound healing, dermal fibroblasts migrate into the wound and produce a newly deposited ECM mainly constituted by collagen and fibronectin^64,68^. These fibrous structures represent the scaffold for the subsequent epithelium migration, and fibre orientation, in particular, is critical e.g. for scarring^68,69^. Similarly, in the case of cancer, aligned collagen fibres around solid tumours favour cell migration and invasion of neighbouring tissue^70^. This study indicates that the smooth topographical confinement in vitro mimics the interaction between cells and the ECM, imposing spatial constraints on the formation and maturation of focal adhesions and on the consequent development of directional tension^6,67,71,72^. Moreover, based on our data, we propose that ROCK pathway plays a fundamental role in the topography sensing of complex multicellular systems. ROCK signalling most likely affects the balance between cellular tension and actin cortex deformability, thereby confining the cellular contacts to the topographic features. One can speculate that in this topographical condition focal adhesions can form only to the ridges and this induces the observed cellular orientation via cytoskeleton reorganization. Even though we cannot completely rule out the effects of cell–cell contacts in this process, we observed that the intercellular junctions are not necessary in the directional migration of cell collectives on microtopographies. This further suggests that the substrate topography induces single cells to develop local directional tension and each individual cell is self-propelled following the contact guidance.

## Conclusions

The tool presented here, based on photopatterning of azobenzene-containing thin films, allowed us to identify the microtopographical pattern that most efficiently provoked coherent and fast migration of epithelium. These topographies had 1 µm periodicity, whose size correlates well with natural dimensions of type I collagen fibres. We note that, in comparison to often-used lithographic approaches, interference lithography on azobenzene-containing materials generates smooth, low aspect ratio sinusoidal patterns. These patterns can be produced with periodicities in the biologically relevant range (300 nm–5 µm) in just one step. Our experiments also indicate that epithelial monolayers behave analogously to nematic liquid crystals. Alike micro-features on a polymer substrate can orient liquid crystalline domains, photoinscribed SRG can organize the cooperative motion of epithelial cell sheets by controlling their anchoring points. In fact, the surface topography was able to induce orientation and elongation of cells and their collective migration was directed by the topographical cues also when cell–cell junctions were disrupted. This work represents an original example of the use of photosensitive substrate based on a new class of azobenzene-containing materials, i.e. molecular glasses, for the study of collective cell motion. These materials open up new avenues for real-time control of the progression of multicellular system movement, due to their possibility to be manipulated with light in a reversible, remotely controllable, and non-invasive way. The used material is capable of being reshaped by light in real time underneath adherent cells^73^. This unique property provides a wealth of possibilities for further studies on the dynamic modulation of cell-material interaction during collective migration, showing the potential of azobenzene-based materials for new applications, such as smart bandages for wound healing and in vitro tools for the study of cancer plasticity.

## Supplementary information

Supplementary Information.

Supplementary Video 1.

Supplementary Video 2.

Supplementary Video 3.

Supplementary Video 4.

Supplementary Video 5.
