# Supplementary material for: Azobenzene-based sinusoidal surface topography drives focal adhesion confinement and guides collective migration of epithelial cells
Source: Sci Rep. 2020 Sep 18;10:15329. doi: 10.1038/s41598-020-71567-w (PMC7501301; doi:10.1038/s41598-020-71567-w)
Supplement: Supplementary file 1 — Supplementary Information. [file 41598_2020_71567_MOESM1_ESM.docx]

Supporting Information

Azobenzene-based sinusoidal surface topography drives focal adhesion confinement and guides collective migration of epithelial cells

Chiara Fedele ^a^, Elina Mäntylä ^b^, Brian Belardi ^c^, Tiama Hamkins-Indik ^c^, Silvia Cavalli ^d^, Paolo A. Netti ^d^, Daniel A. Fletcher ^c, e, f^ Soile Nymark ^b^, Arri Priimagi ^a, ¶,^ * and Teemu O. Ihalainen ^b, ¶^ *

^a^ Faculty of Engineering and Natural Sciences Tampere University, Tampere, Finland

^b^ BioMediTech and Faculty of Medicine and Health Technology, Tampere University, Tampere, Finland

^c^ Department of Bioengineering and Biophysics Program, University of California, Berkeley, CA, 94720 USA

^d^ Istituto Italiano di Tecnologia, Center for Advanced Biomaterials for Health Care @CRIB

^e^ Chan Zuckerberg Biohub, San Francisco, CA 94158

^f^ Division of Biological Systems and Engineering, Lawrence Berkeley National Laboratory, Berkeley, CA, 94720 USA

^¶^ Equal contribution

* Correspondence to teemu.ihalainen@tuni.fi or arri.priimagi@tuni.fi

Table of Contents

| Figure S1 | DR1-glass chemical structure, UV/Vis absorption spectrum of DR1-glass and Diffraction efficiency during photopatterning. | p. 3 |
| --- | --- | --- |
|  | Immunostaining procedure | p. 3 |
| Figure S2 | Immunostaining of mature epithelium. | p. 3 |
| Figure S3 | AFM analysis of collagen coating. | p. 4 |
| Figure S4 | Alignment analysis of cell colonies during growth. | p. 5 |
| Figure S5 | Focal adhesion distribution during cell growth. | p. 6 |
| Figure S6 | Distribution of an isotropic fluorescence signal. | p. 6 |
|  | Cell migration in presence of pharmacological inhibitors of ROCK or PI(3)K pathways. | p. 7 |
| Figure S7 | Cell migration in presence of pharmacological inhibitors of ROCK or PI(3)K pathways. | p. 8 |
| Figure S8 | Confocal image of a rear area of the wound. Vinculin and pFAK immunostainings. | p.9 |
| Figure S9 | Spatial correlation function of the v component. | p.9 |
| Figure S10 | MDCK wild type cells cultured in low Ca^2+^ conditions. | P.10 |
| Figure S11 | Characterization of MDCK ZO-1/ZO-2 KO cells. | P.11 |
| Figure S12 | DECMA1- anti E-cadherin antibody essay during wound healing. | p.12 |
|  | References | p.12 |


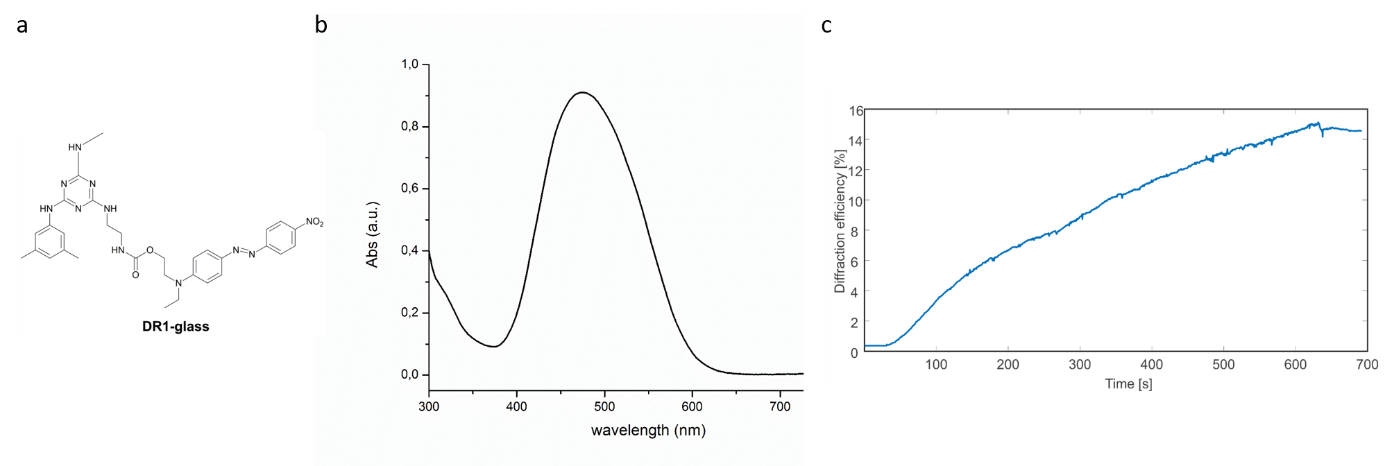


**Figure S1. DR1-glass characteristics.** **a,** DR1-glass chemical structure, **b**, UV/Vis absorption spectrum of DR1-glass, and **b**, diffraction efficiency during photopatterning.

**Immunostaining**

Cells in Figure S2 were fixed with 4 % paraformaldehyde for 15 min, permeabilized with 0.1 % Triton X-100 in PBS for 15 min and then immunolabeled with either mouse anti-Lamin A/C (1:200, Abcam #ab8984), or rabbit anti-ZO-1 (1:50, Abcam #ab59720). These were combined with anti-mouse Alexa-488 (1:200, Thermo Fisher #A32723) or 568-linked secondary antibody (1:200, Thermo Fisher #A10037). Atto-633-phalloidin (1:50, Sigma #68825) was used to label the actin cytoskeleton. DAPI stain for nuclei was in the ProLong Gold antifade mountant (Thermo Fisher, #P36935).


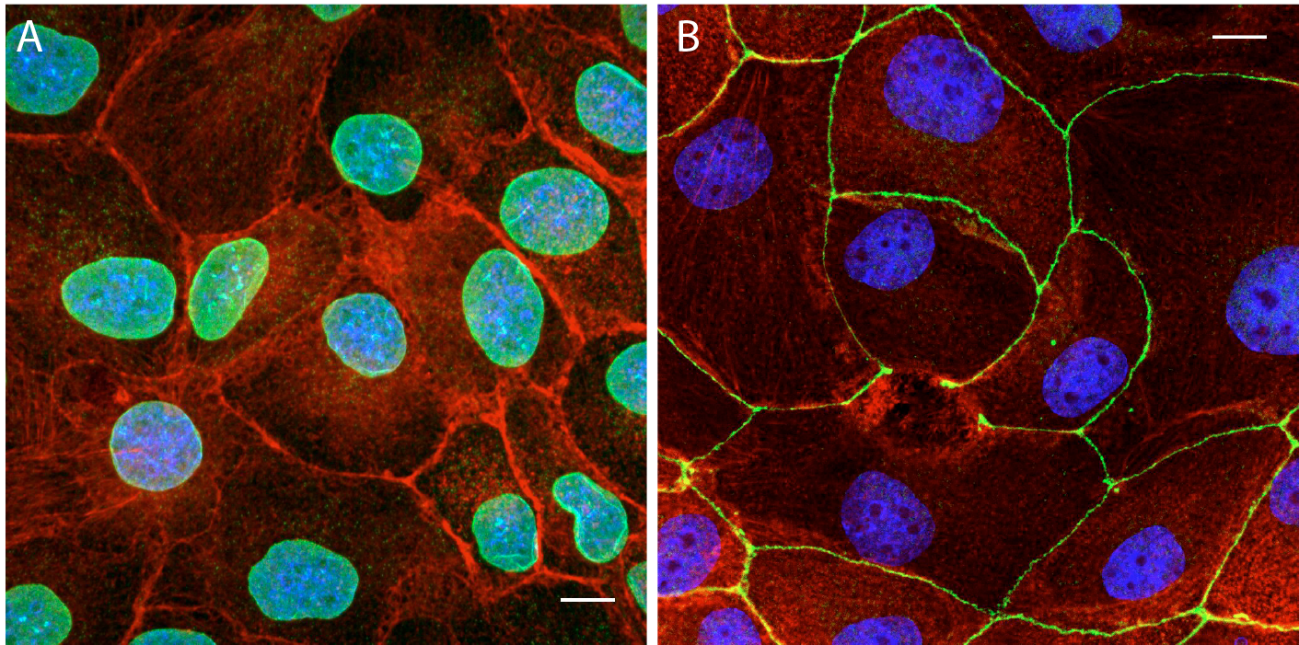


**Figure S2. Immunolabeled cell monolayer on a flat DR1-glass surface.** Representative image of MDCK wt cells with DAPI stained nuclei (blue) and phalloidin labelled actin cytoskeleton (red) showing equal morphology of the monolayers grown on a) flat or on b) DR1-glass. The green depicts a) Lamin A/C, and b) ZO-1. Scale bars, 10 µm.


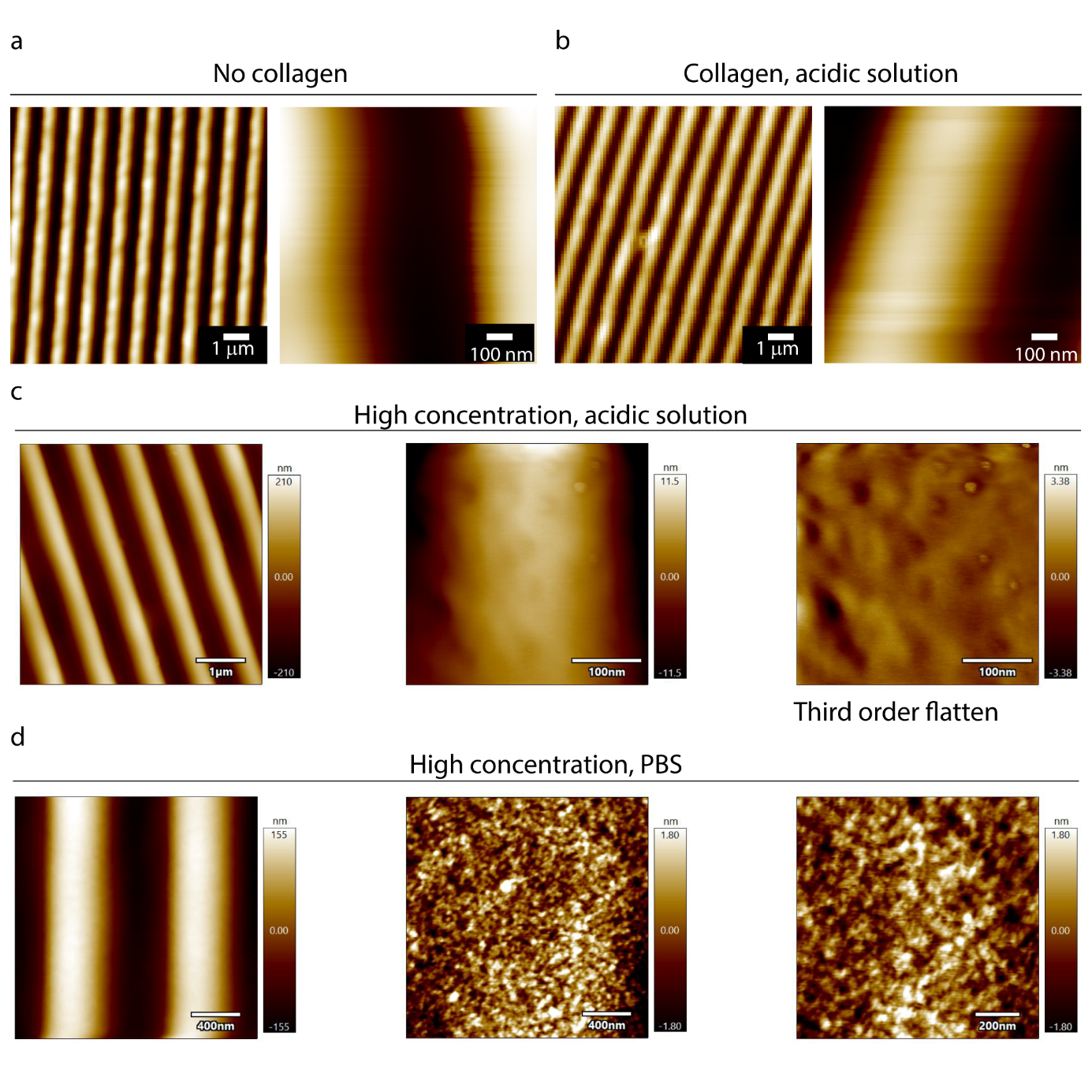


**Figure S3. AFM analysis of collagen coating.** SRG on DR1-glass **a**, without any collagen coating **b**, in presence of collagen I coating (50 µg/ml from acetic acid solution) **c**, with a higher concentration of collagen I from acidic solution and **d**, from PBS. The AFM analysis revealed that in all the presented concentrations, the microtopography is not hidden. Furthermore, a porous structure of the collagen is identified when deposited in higher concentrations, whose texture depends on the solution pH.


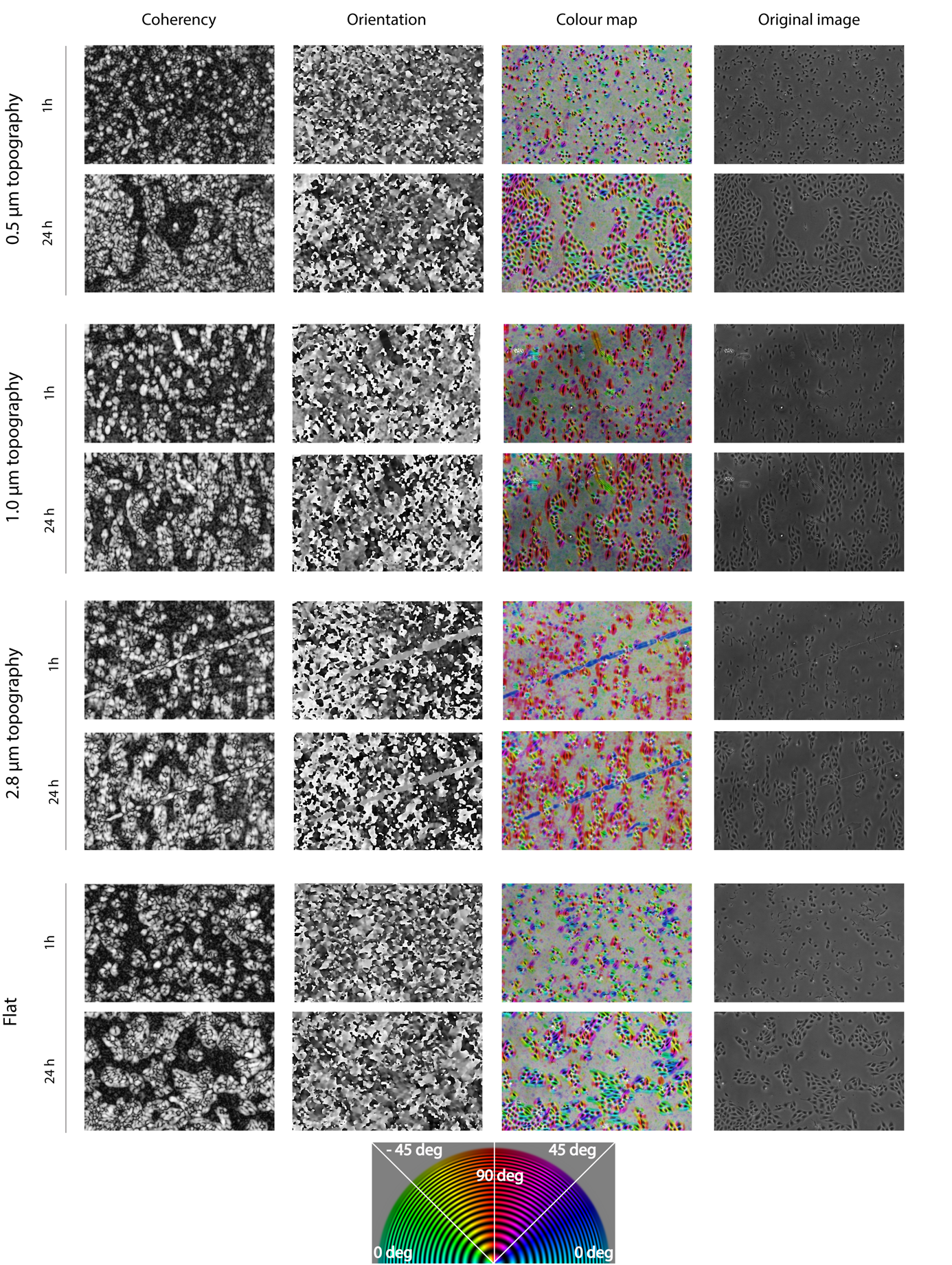


**Figure S4. Alignment analysis of cell colonies during growth.** Local maps of the morphological parameters of coherency and orientation as calculated by OrientationJ. In the colour map the hue represents the orientation angle, whereas the saturation indicates the coherency, and the brightness represents the original image.


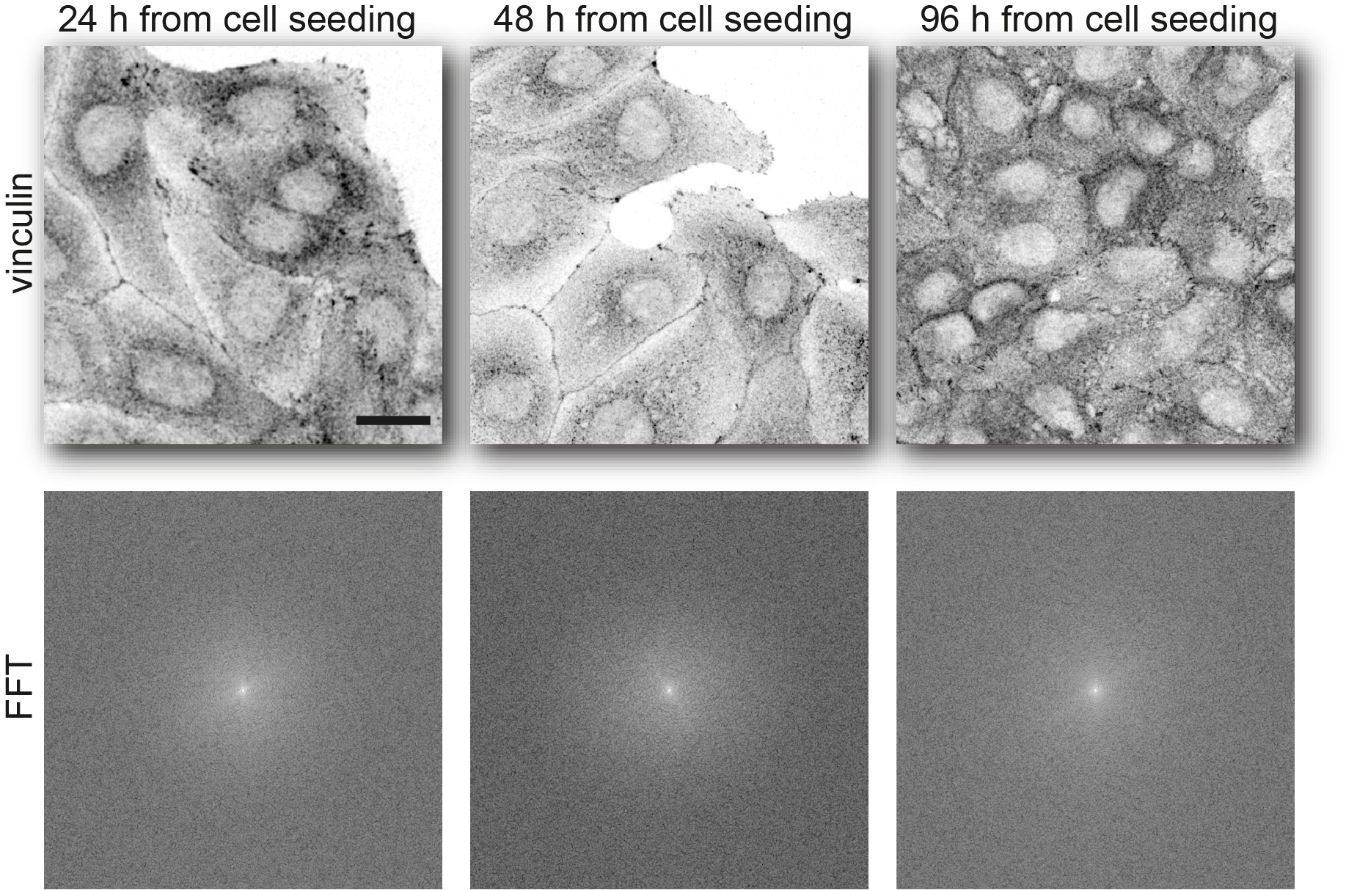


**Figure S5. Focal adhesion distribution during cell growth.** FFT analysis of focal adhesion distribution as depicted by vinculin staining on cells growing on flat DR1-glass samples for 24 – 96 h. The scale bar, 20 µm.


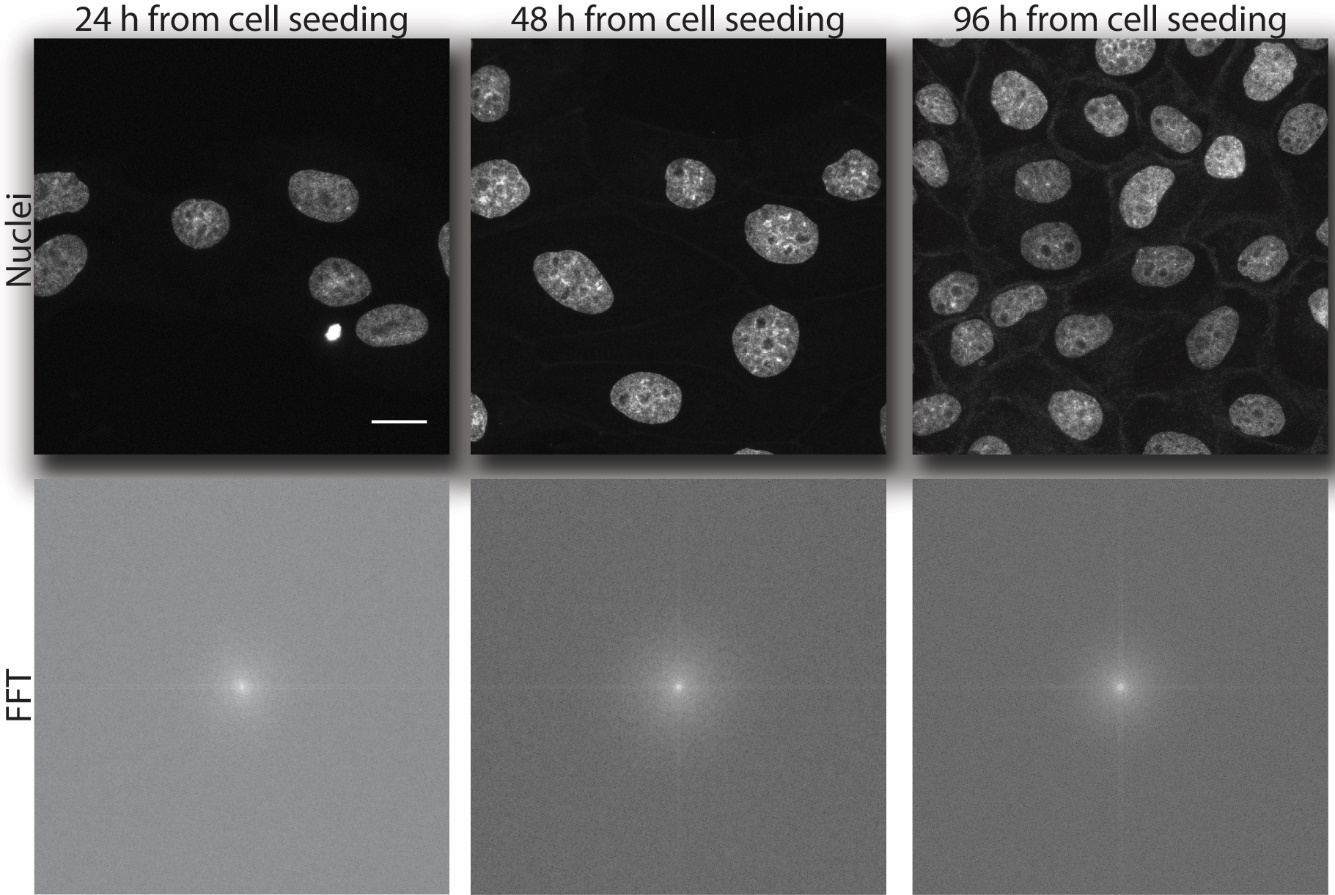


**Figure S6. Distribution of an isotropic fluorescence signal.** FFT analysis of DAPI nuclei staining as control for a non-periodic signal.

**Cell migration in presence of pharmacological inhibitors of ROCK or PI(3)K -pathways.**

For the perturbation of the signalling pathways related to cell migration, ROCK inhibitor (Y-27632, 5 μM, Sigma-Aldrich #SCM075) and PI(3)K inhibitor (LY294002, 10 μM, Sigma-Aldrich) were added to the culture medium before the migration experiment.

It is well-established that the cellular actomyosin machinery is important in determining cell stiffness.^1^ This activity is regulated by the phosphorylation of the myosin light chain (MLC) through MLC kinase, which is triggered by elevated levels of the small GTPase RhoA and subsequent upregulation of RhoA-dependent kinase (ROCK).^2,3^ The activation of another small GTPase, Rac1, is generally accompanied by elevated levels of phosphatidylinositol-3-kinase (PI(3)K) and induces the formation of membrane ruffles and protrusions.^3^ By selectively inhibiting these signalling pathways (with ROCK-1/2 inhibitor Y-27632 and PI(3)K inhibitor LY294002), we were able to manipulate the balance between cellular contractility and membrane protrusions (Fig. S7a).^4^

The PI(3)K inhibition yields relative increase in tension of the actin cortex near the cell membrane and reduces the membrane ruffling. Consequently, the cells can adhere only on the ridges of the pattern, as already shown for single cancer cells on micro-posts.^4^ Indeed, when we seeded the cells on 1 µm patterns and inhibited the PI(3)K signalling pathway, the cell colonies oriented themselves strongly to the pattern direction. The coherency of the cell colonies was even higher in PI(3)K inhibition than in the control sample (Fig. S7b and S7c, Supplementary movie 3). As opposed to PI(3)K inhibition, the ROCK inhibitor selectively lowers the intracellular tension by reducing the myosin II activity. This allows the cells to deform their actin cortex more easily, and the cell membrane to follow the grooved substrate. Consequently, during ROCK-1/2 inhibition the cell colonies showed reduced coherency (Fig. S7b and S7c, Supplementary movie 3).

The selective inhibition of ROCK and PI(3)K pathways strongly influenced the topography sensing of the epithelial colonies. PI(3)K inhibition increased the elongation of the cell colonies along the grooves, whereas ROCK inhibition abolished directional migration. Interestingly, PI(3)K activity has been shown to be fundamental for maintaining an ordered advancement of the epithelial cell monolayer in wound healing.^5^ PI(3)K is also known to be responsible for chemotactic migration and dynamic regulation of integrin activity.^6,7^ On the other hand, ROCK inhibition has been found not to be crucial for the collective migration itself, but for the formation of finger-like structures in the leading edge of an advancing wounded epithelium.^8^ ROCK inhibition has also been found to speed up the migration due to increased adhesion, spreading and ruffling of cells.^9^ The balance between PI(3)K and ROCK-signalling is also pivotal for single cell migration in response to topography density gradients.^10^ In agreement with these observations, we can confirm that ROCK plays a fundamental role in the topography sensing of complex multicellular systems, most likely through balancing cellular tension and actin cortex deformability, thereby dictating the multicellular response on structured surfaces.


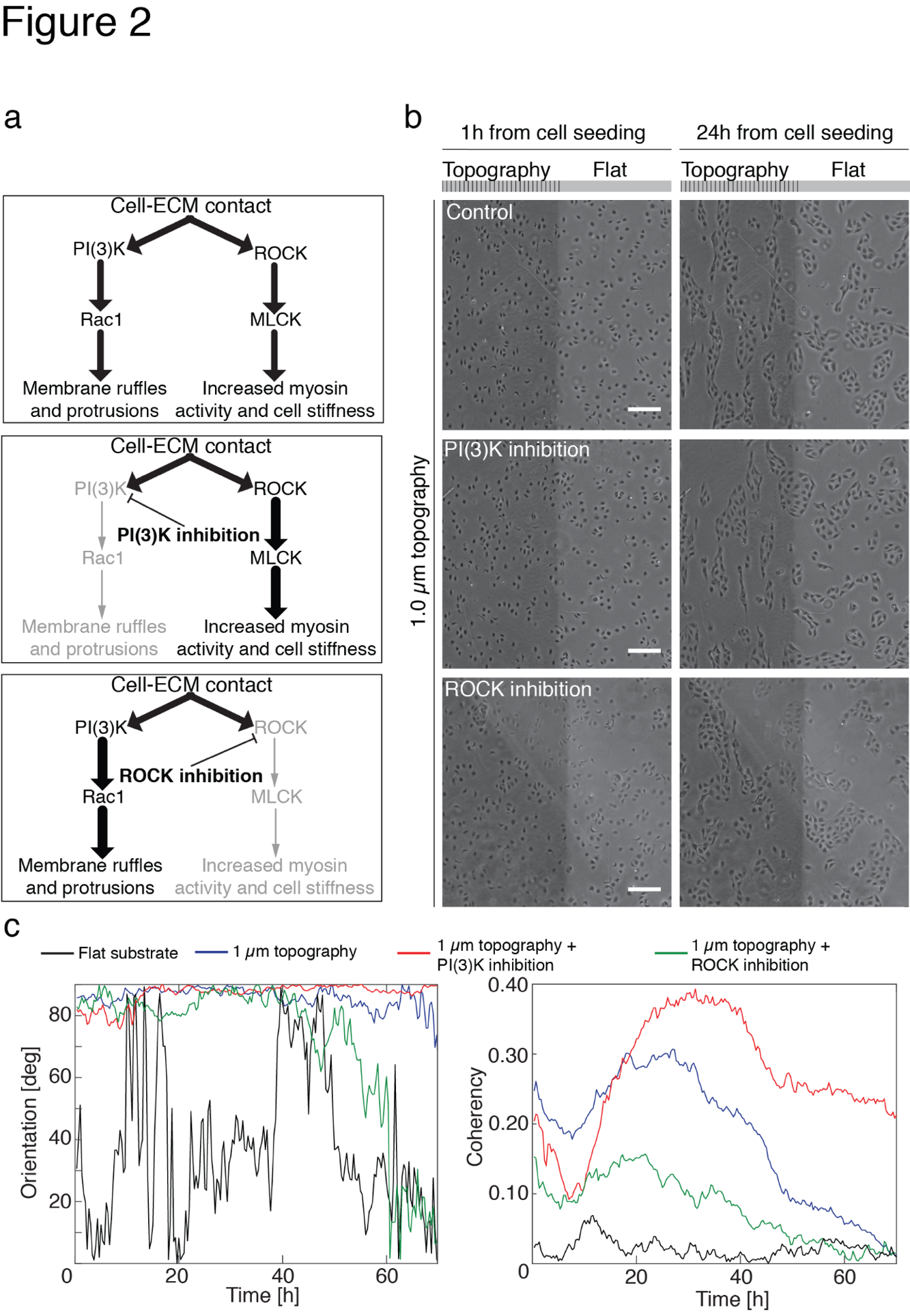


**Figure S7. Cell migration in presence of pharmacological inhibitors of ROCK or PI(3)K pathways.** **a,** Schematic representation of the ROCK/PI(3)K pathways balance. Here, bold lines represent the active signalling pathway, while grey lines represent the pharmacologically inhibited pathways. **b,** Phase contrast images of MDCK cells during migration on patterns with 1 µm pitch in presence of different drugs (the sketch of the topography indicates only where the topography is, bearing no quantitative information, but only illustrative purposes). Control sample does not contain any drugs. The images are taken after 1 h and 24 h of migration. Scale bars are 100 μm. **c,** Orientation and coherency progression during migration.


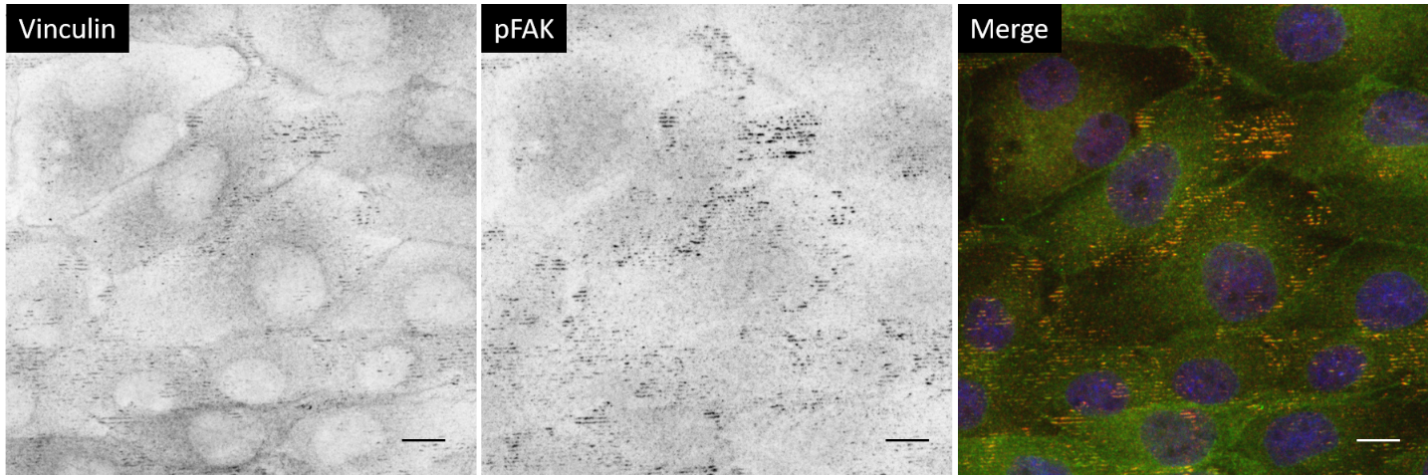


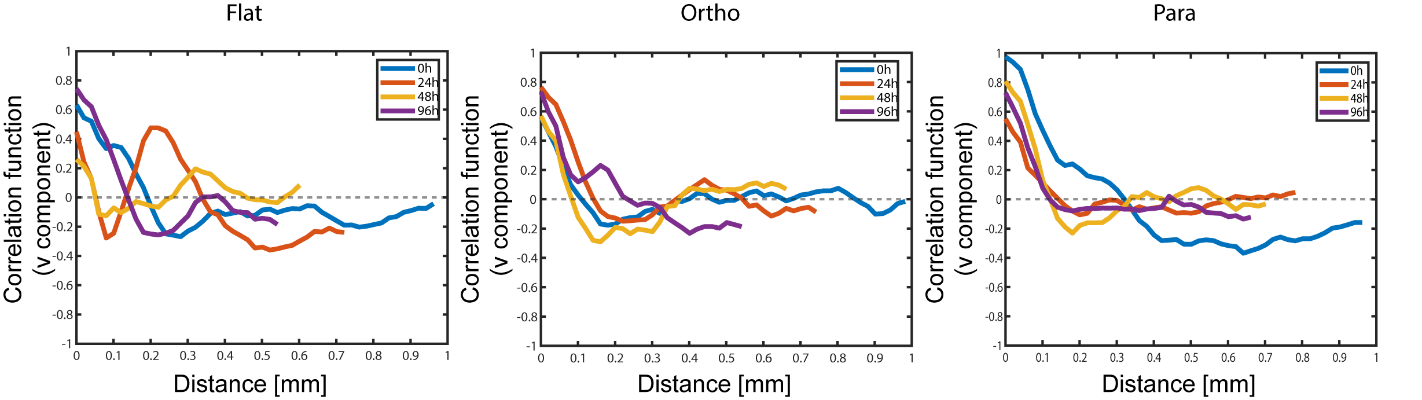
**Figure S8. Focal adhesion distribution in wound healing.** Confocal image of a rear area of the wound of vinculin and pFAK immunostainings. Scale bars are 20 µm.

**Figure S9. Spatial correlation function of the v component during wound healing.** Time evolution of the spatial correlation function of the v component (y direction) of the velocity vectors, as extracted by PIV analysis, for different pattern configurations in wound healing experiments.


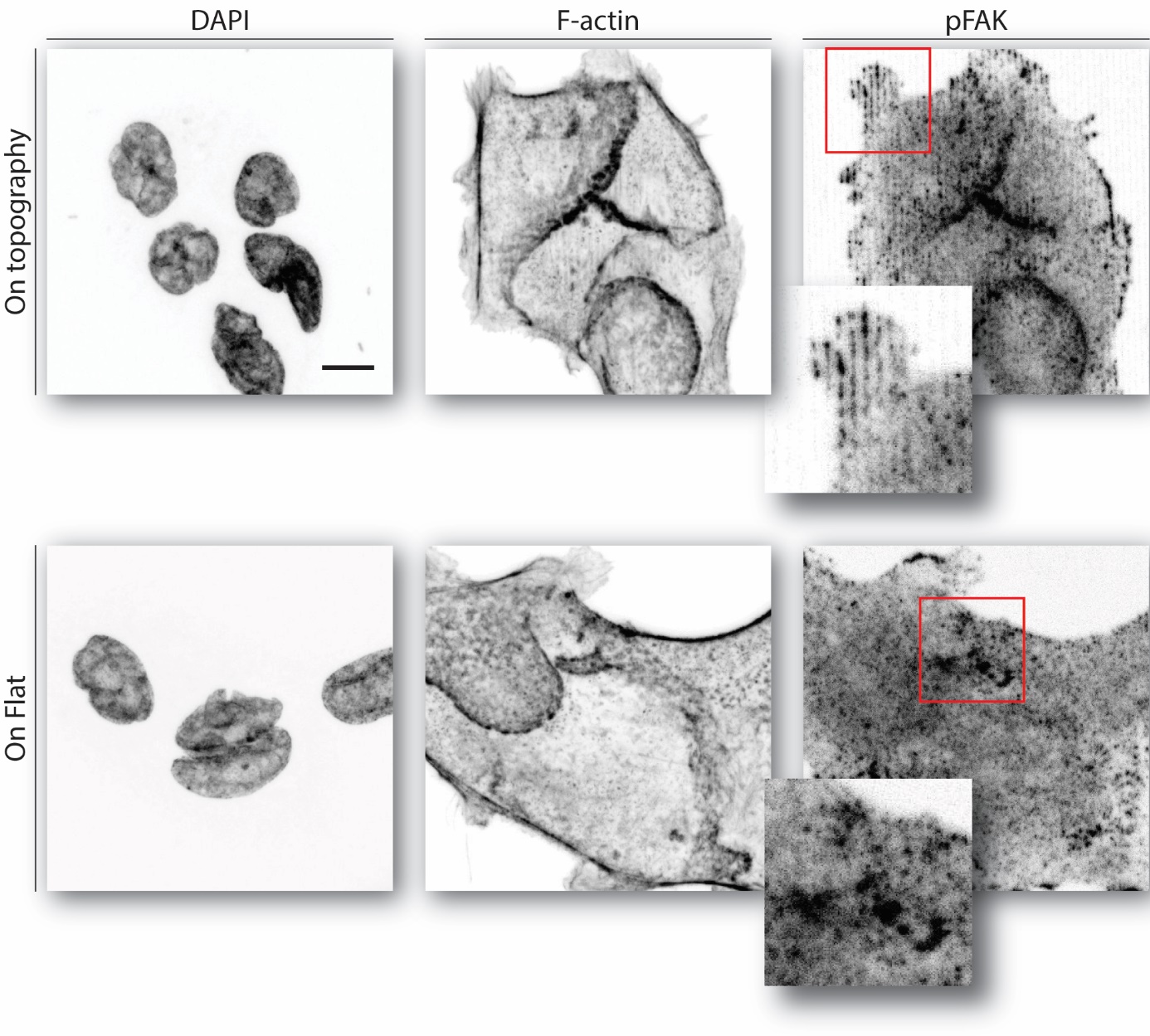
 **Figure S10. MDCK wt cells cultured in low Ca^2+^ conditions.** Cells were cultured on DR1-glass for 1 week in Ca^2+^ depleted medium and then immunostained. Scale bar is 15 µm.


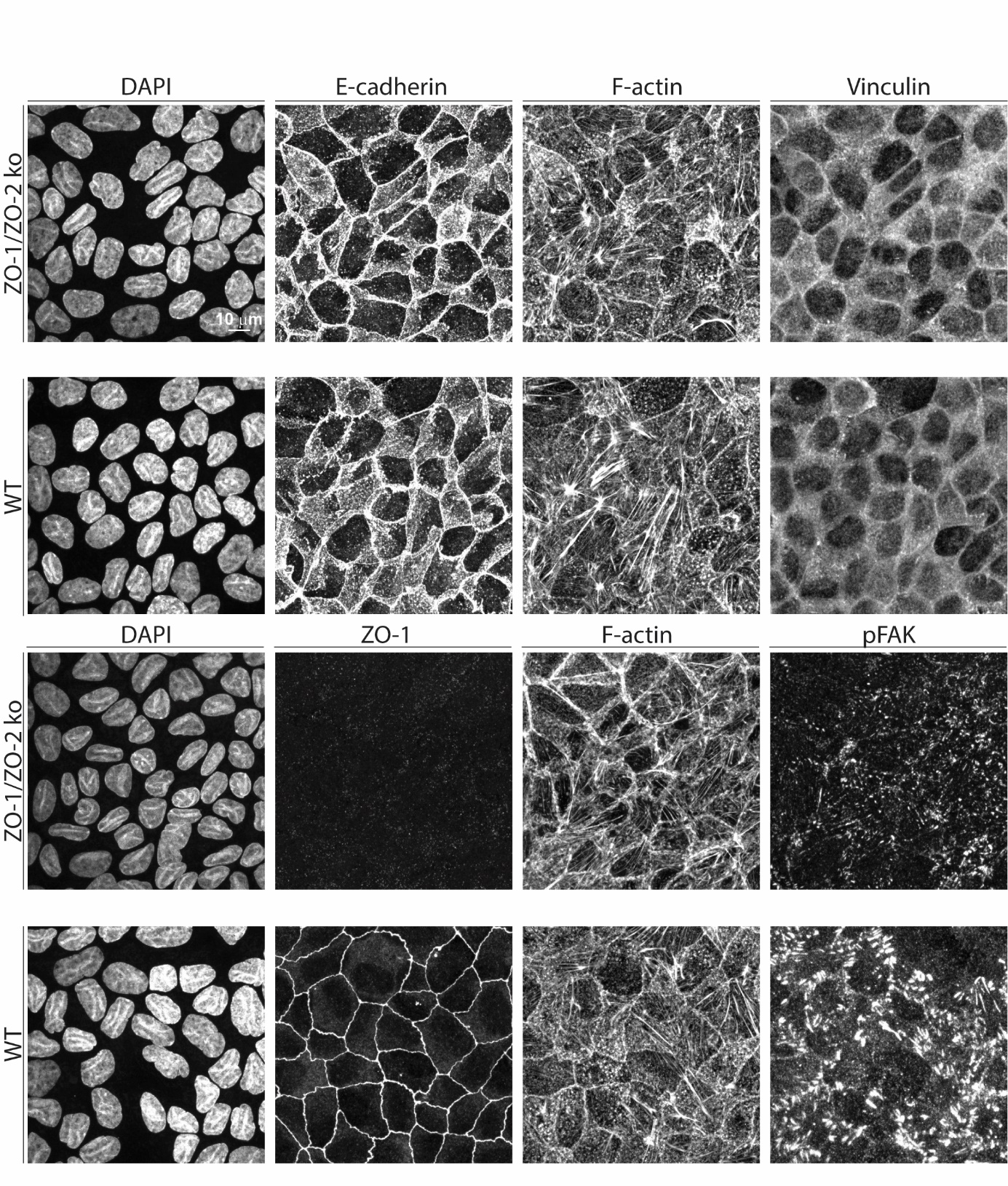
**Figure S11. Characterization of MDCK ZO1/ZO2 knockout cells.** Immunostaining (maximum intensity projection) of E-cadherin, ZO-1, F-actin, vinculin, pFAK, and DNA (DAPI). MDCK ZO-1/ZO-2 knockout cells compared to wild type. Cells were cultured on glass coverslips coated with monomeric rat tail collagen type I for 10 days and fixed at confluency.


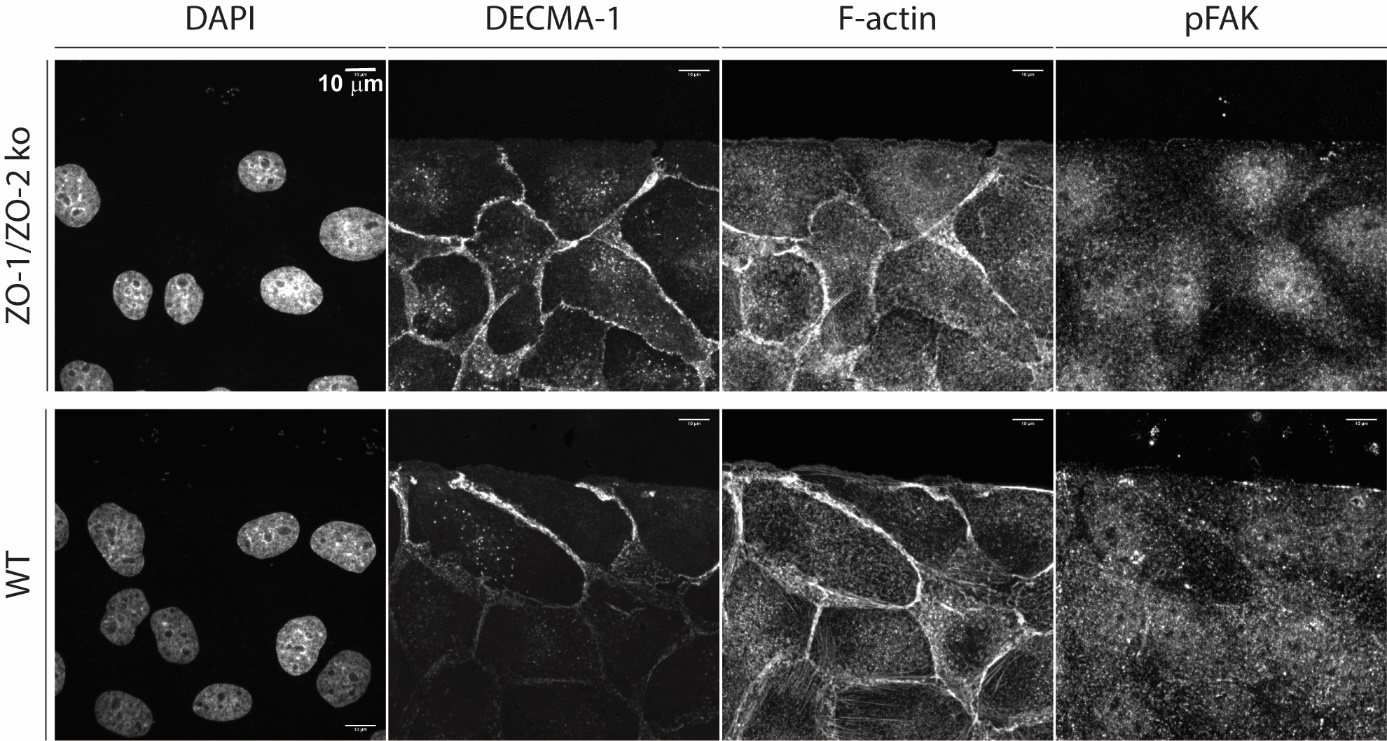
**Figure S12. DECMA1- anti E-cadherin antibody essay during wound healing.** Immunostaining (maximum intensity projection) of DECMA1, F-actin, pFAK, and DAPI right after removal of PDMS stencils both in MDCK ZO-1/ZO-2 KO and wild-type cells.

**References:**

1 Das, T. *et al.* A molecular mechanotransduction pathway regulates collective migration of epithelial cells. *Nat. Cell Biol.* **17**, 276-287, (2015).

2 Riento, K. & Ridley, A. J. Rocks: multifunctional kinases in cell behaviour. *Nat. Rev. Mol. Cell Biol.* **4**, 446-456, (2003).

3 Wettschureck, N. & Offermanns, S. Rho/Rho-kinase mediated signaling in physiology and pathophysiology. *J. Mol. Med.* **80**, 629-638, (2002).

4 Park, J. *et al.* Directed migration of cancer cells guided by the graded texture of the underlying matrix. *Nat. Mater.*, (2016).

5 Marmaras, A. *et al.* Topography-mediated apical guidance in epidermal wound healing. *Soft Matter* **8**, 6922-6930, (2012).

6 Kim, H. N., Hong, Y., Kim, M. S., Kim, S. M. & Suh, K.-Y. Effect of orientation and density of nanotopography in dermal wound healing. *Biomaterials* **33**, 8782-8792, (2012).

7 Lauffenburger, D. A. & Horwitz, A. F. Cell migration: a physically integrated molecular process. *Cell* **84**, 359-369, (1996).

8 Provenzano, P. P. *et al.* Collagen reorganization at the tumor-stromal interface facilitates local invasion. *BMC medicine* **4**, 38, (2006).

9 Yamaguchi, N., Mizutani, T., Kawabata, K. & Haga, H. Leader cells regulate collective cell migration via Rac activation in the downstream signaling of integrin β1 and PI3K. *Sci. Rep.* **5**, 7656, (2015).

10 Devreotes, P. & Horwitz, A. R. Signaling networks that regulate cell migration. *Cold Spring Harb. Perspect. Biol.* **7**, a005959, (2015).
